# Joint trajectories of nutritional risk and fluid balance and prognosis in critically ill adults: a dual-trajectory modeling study using MIMIC-IV and eICU

**DOI:** 10.3389/fnut.2026.1881675

**Published:** 2026-07-20

**Authors:** Yang He, Jiali Huang, Xidong Wang, Gaosheng Zhou, Jinglan Liu

**Affiliations:** 1 Yichang Central Department of Critical Care Medicine, The First College of Clinical Medical Science, Yichang Central People's Hospital, China Three Gorges University, Yichang, Hubei, China; 2 Yichang Central People's Hospital, Yichang, Hubei, China; 3 Yichang Clinical Research Center for Sepsis, Yichang Central People's Hospital, Yichang, Hubei, China; 4 Hubei Provincial Clinical Research Center for Critical Care Medicine (Sepsis Research Collaborative Unit), Yichang Central People's Hospital, Yichang, Hubei, China

**Keywords:** critical care, eICU, fluid balance, GNRI, group-based multi-trajectory model, MIMIC-IV, mortality, nutritional risk

## Abstract

**Background:**

Nutritional risk and fluid balance evolve rapidly during critical illness, but their joint longitudinal patterns and prognostic relevance remain incompletely characterized. This study identified early joint trajectories of GNRI-derived nutritional risk and fluid balance and evaluated their associations with mortality in critically ill adults.

**Methods:**

This retrospective dual-database study used MIMIC-IV as the development cohort and eICU as an independent reproducibility cohort. Adult ICU patients with an ICU stay >=3 days and at least three paired daily measurements of GNRI-derived nutritional risk and weight-standardized fluid balance during ICU days 1–7 were included. Nutritional risk was defined as 98 minus daily GNRI. Group-based multi-trajectory modeling identified joint phenotypes. Multivariable logistic and Cox regression models evaluated associations with hospital and short-term mortality. Sensitivity analyses included landmark analyses, extended covariate adjustment, proportional-hazards diagnostics, posterior-classification assessment, and incremental prediction evaluation.

**Results:**

The analysis included 1,243 patients from MIMIC-IV and 9,912 from eICU. Three reproducible joint trajectory phenotypes were identified: persistent moderate-to-high nutritional risk with gradual deresuscitation (Group A), initially severe nutritional risk with high early fluid load and rapid decline (Group B), and worsening nutritional risk with mild-to-moderate deresuscitation (Group C). Group B had the highest crude mortality in both cohorts, with hospital mortality of 40.0% in MIMIC-IV and 34.7% in eICU. In fully adjusted models, Group B was associated with higher 30-day mortality in MIMIC-IV (HR 1.59, 95% CI 1.12–2.28) and higher hospital mortality in eICU (OR 1.95, 95% CI 1.55–2.45). Group C was also associated with increased 90-day mortality in MIMIC-IV (HR 1.37, 95% CI 1.10–1.72) and hospital mortality in eICU (OR 1.29, 95% CI 1.14–1.46).

**Conclusion:**

Early joint trajectories of GNRI-derived nutritional risk and fluid balance identified reproducible prognostic phenotypes in selected critically ill adults with sufficient repeated measurements. The phenotype combining severe early nutritional risk with high initial fluid load showed the highest mortality risk. Incremental prediction analyses indicated modest additional prognostic information beyond baseline GNRI and early fluid balance, supporting dynamic nutrition-fluid phenotyping as a complement to conventional severity assessment.

## 1 Introduction

Critical illness profoundly disrupts nutritional metabolism and fluid homeostasis. Systemic inflammation, neuroendocrine stress, immobility, organ dysfunction, and interruptions in enteral feeding accelerate protein catabolism and loss of lean body mass, while simultaneously altering vascular permeability, renal handling of sodium and water, and the distribution of intravascular and interstitial fluid. Contemporary critical care nutrition guidelines emphasize early recognition of nutritional risk and individualized nutrition support in patients who are unable to maintain adequate oral intake (1, 2). Nevertheless, nutritional assessment in the intensive care unit (ICU) remains challenging because conventional markers such as serum albumin and body weight are strongly influenced by inflammation, capillary leak, edema, and fluid therapy rather than reflecting nutritional status alone (3).

Several approaches have been proposed to quantify nutritional risk in critically ill patients. The Nutrition Risk in Critically ill (NUTRIC) score was developed to identify ICU patients most likely to benefit from aggressive nutrition therapy, but its calculation requires severity and inflammatory variables that may not be uniformly available in large clinical databases (4). The Geriatric Nutritional Risk Index (GNRI), calculated from serum albumin and the ratio of actual to ideal body weight, is simple, reproducible, and widely used as a prognostic nutritional index (5). Although originally developed for older medical patients, GNRI has been increasingly applied in acute and critical care settings because its components are routinely captured in electronic health records. However, both albumin concentration and body weight can change dynamically during critical illness as a consequence of inflammation, fluid administration, diuresis, and renal replacement therapy. Therefore, a single baseline GNRI value may inadequately represent the evolving nutritional risk profile of critically ill patients.

Fluid balance is another fundamental determinant of outcome in critical illness. Adequate early fluid resuscitation may restore organ perfusion, whereas persistent positive fluid balance and fluid overload can worsen pulmonary edema, impair oxygenation, increase venous congestion, and contribute to renal and multiorgan dysfunction. A systematic review and meta-analysis of observational studies reported that fluid overload and positive cumulative fluid balance were associated with increased mortality among adult critical care patients (6). Conservative fluid management or deresuscitation strategies after the resuscitation phase have also been associated with improved fluid-related outcomes in sepsis and acute respiratory distress syndrome populations (7). Similarly, cumulative fluid balance has been linked to mortality and longer duration of mechanical ventilation in acute respiratory distress syndrome (8). However, most prior studies have summarized fluid status using isolated time points, peak values, or cumulative balance, which may obscure clinically important patterns of early resuscitation, stabilization, and deresuscitation.

Nutritional risk and fluid balance are biologically interdependent during critical illness. Fluid accumulation can dilute serum albumin, increase measured body weight, and mask nutritional depletion, whereas malnutrition, hypoalbuminemia, reduced muscle mass, and systemic inflammation can increase susceptibility to edema and impaired fluid mobilization. Nutritional therapy itself may also contribute to total daily fluid input through enteral or parenteral formulations. This reciprocal relationship implies that the prognostic effect of nutritional risk may depend on concurrent fluid balance patterns, and conversely, the clinical implications of fluid balance may differ according to the patient's nutritional reserve. Most available studies have evaluated nutritional risk or fluid balance separately, leaving the joint dynamic phenotype of these two domains poorly characterized.

Trajectory-based methods provide a framework for addressing this gap. Group-based trajectory modeling identifies latent subgroups of individuals who follow similar longitudinal patterns, thereby enabling clinically interpretable phenotyping beyond static baseline measurements (9). Group-based multi-trajectory modeling further extends this approach by jointly modeling multiple repeated indicators and identifying subgroups that share similar patterns across more than one clinical domain (10). In critical care research, fluid balance trajectory studies have shown that time-dependent patterns of fluid administration and removal are associated with clinical outcomes in septic shock and in patients receiving renal replacement therapy (11, 12). A recent Frontiers in Nutrition study also demonstrated that fluid balance trajectories were associated with short-term prognosis among older malnourished ICU patients using MIMIC-IV data (13). However, that work focused on a single fluid-balance domain, restricted the population to older patients with malnutrition, and did not evaluate external reproducibility in an independent multicenter ICU database.

This dual-database study used MIMIC-IV as the development cohort and the eICU Collaborative Research Database as an independent reproducibility cohort (14, 15). The study identified joint trajectories of nutritional risk and fluid balance during the first 7 days after ICU admission among adult critically ill patients and evaluated their associations with hospital mortality and short-term survival. The central hypothesis was that group-based multi-trajectory modeling would identify reproducible nutritional-fluid phenotypes with distinct prognostic profiles and provide complementary prognostic information beyond isolated baseline nutritional indices or static fluid balance measures.

## 2 Materials and methods

### 2.1 Data sources

This retrospective observational study used two publicly available critical care databases. The Medical Information Mart for Intensive Care IV (MIMIC-IV), which contains comprehensive ICU data collected between 2008 and 2022, and the eICU Collaborative Research Database (eICU-CRD), which includes ICU admissions from multiple US centers during 2014–2015. Access to both databases was obtained after completion of the required data use training (certification No. 13278787).

### 2.2 Study population

Adult ICU patients aged 18 years or older were screened. For patients with multiple ICU records, the first eligible ICU stay was retained according to database-specific identifiers. Patients were excluded if they had missing hospital mortality information, ICU length of stay less than 3 days, unavailable sex, height, or body weight, fewer than three available daily GNRI measurements during ICU days 1–7, fewer than three available daily fluid balance measurements during ICU days 1–7, or fewer than three paired daily GNRI-fluid balance observations during ICU days 1–7.

### 2.3 Nutritional risk assessment

Daily GNRI was calculated using serum albumin, actual body weight, and ideal body weight: GNRI = 1.489 x albumin (g/L) + 41.7 x (actual body weight / ideal body weight). When actual body weight exceeded ideal body weight, the ratio was set to 1. Ideal body weight was calculated using the Lorentz formula. For trajectory modeling, nutritional risk was defined as 98 minus daily GNRI, so that higher values represented greater nutritional risk. Baseline nutritional risk was defined as the first available nutritional risk score during ICU days 1–3.

### 2.4 Fluid balance assessment

Daily fluid balance was calculated as total fluid input minus total fluid output and standardized by baseline body weight (mL/kg/day). In MIMIC-IV, daily fluid balance was calculated from input and output events extracted from ICU records. In eICU, because total fields in the intake-output table may represent cumulative or repeated total values, the primary eICU analysis used cell-level input and output records to calculate daily fluid balance. Corrected netTotal-based fluid balance was retained for sensitivity analyses. Cumulative fluid balance and fluid overload percentage were also calculated for descriptive and supplementary analyses.

### 2.5 Outcomes and covariates

The primary outcome was hospital mortality. Secondary outcomes included 30-day and 90-day mortality in MIMIC-IV and 30-day in-hospital mortality in eICU. Baseline covariates included age, sex, body mass index, comorbidities, mechanical ventilation, renal replacement therapy, diuretic use, and norepinephrine use. Disease severity was adjusted using SOFA score and Charlson Comorbidity Index in MIMIC-IV and APACHE score in eICU, reflecting the availability and completeness of database-specific severity scores. Variables with substantial missingness were not included in the primary multivariable models.

### 2.6 Joint trajectory modeling

Group-based multi-trajectory modeling was used to identify joint longitudinal patterns of nutritional risk and fluid balance over ICU days 1–7. Models were fitted with the multlcmm function from the lcmm package in R, using linear link functions for both longitudinal outcomes, random intercepts, and cubic polynomial terms for ICU day in the primary day 1–7 model. Candidate models with one to five latent classes were evaluated. For multi-class models, multiple random starts were implemented through grid search to reduce convergence to local maxima. MIMIC-IV was used to derive candidate trajectory structures, and eICU was used to evaluate external reproducibility of trajectory patterns and prognostic associations without directly applying a pre-fitted MIMIC-IV model. Model selection considered convergence status, AIC, BIC, entropy, average posterior probability (AvePP), odds of correct classification (OCC), group size, visual separation of trajectories, and clinical coherence. A three-class model was selected as the primary model because it provided a parsimonious, clinically interpretable structure that was broadly reproduced in eICU. Candidate model-selection diagnostics, paired-observation distributions, and posterior classification diagnostics are reported in Supplementary Table S3. Four-class models were retained as sensitivity analyses to explore smaller high-risk phenotypes (Supplementary Table S4 and Supplementary Figure S4).

### 2.7 Statistical analysis

Continuous variables were summarized as median and interquartile range or mean and standard deviation, as appropriate, and categorical variables were summarized as counts and percentages. Logistic regression models were used for hospital mortality and eICU 30-day in-hospital mortality. Cox proportional hazards models were used for 30-day and 90-day mortality in MIMIC-IV, and the proportional hazards assumption was assessed using Schoenfeld residuals (Supplementary Table S12). Four sequential models were constructed: Model 1 was unadjusted; Model 2 adjusted for age and sex; Model 3 additionally adjusted for body mass index and database-specific severity measures; and Model 4 further adjusted for chronic kidney disease, heart failure, sepsis, mechanical ventilation, renal replacement therapy, furosemide, spironolactone, and norepinephrine use. Patients were assigned to the most likely trajectory group for primary outcome modeling; posterior classification diagnostics and a high-confidence posterior-probability sensitivity analysis were used to evaluate the potential effect of classification uncertainty. Kaplan-Meier curves and log-rank tests were used to compare survival across trajectory groups. Prespecified subgroup analyses were performed according to age, sex, baseline GNRI category, illness severity, sepsis, chronic kidney disease, heart failure, mechanical ventilation, renal replacement therapy, and norepinephrine use. Interaction terms were evaluated using likelihood ratio tests. Restricted cubic spline analyses were performed as supplementary dose-response analyses for baseline nutritional risk and mean daily fluid balance.

Selection bias related to the requirement for sufficient repeated nutritional-fluid measurements was evaluated by comparing included and excluded patients within each database. Exposure-window-related and immortal time bias were examined using landmark sensitivity analyses. A strict day-7 landmark analysis included patients who were alive and still hospitalized at ICU day 7, with follow-up beginning after the landmark. Short-window joint trajectories were also reconstructed using ICU days 1-3, followed by a day-3 landmark analysis among patients alive and still hospitalized at ICU day 3.

Potential construct overlap between GNRI-derived nutritional risk and fluid balance was evaluated using extended adjustment analyses. The fully adjusted Model 4 was further adjusted for baseline GNRI, baseline albumin, day-1 fluid balance, mean fluid balance during ICU days 1–3, cumulative fluid balance during ICU days 1–3, and a combined set including baseline GNRI, baseline albumin, day-1 fluid balance, and cumulative fluid balance during ICU days 1–3. Continuous additional covariates were standardized before model fitting.

Incremental prediction analyses were conducted to evaluate whether joint trajectory groups added prognostic information beyond conventional covariates, baseline GNRI, and early fluid balance. Six logistic prediction models were compared for fixed-time binary outcomes: base covariates alone; base covariates plus baseline GNRI; base covariates plus early fluid balance; base covariates plus baseline GNRI and early fluid balance; base covariates plus joint trajectory group; and the full model including base covariates, baseline GNRI, early fluid balance, and joint trajectory group. Predictive performance was evaluated using 5-fold cross-validated predicted probabilities, AUC, Brier score, calibration intercept and slope, decision curve analysis, integrated discrimination improvement (IDI), and continuous net reclassification improvement (NRI). These analyses used complete-case datasets containing all variables required for model comparison. A high-confidence posterior classification sensitivity analysis restricted to patients with posterior probability >= 0.70 was also performed. All tests were two-sided, and *P* < 0.05 was considered statistically significant. Subgroup analyses were considered exploratory, and no multiplicity adjustment was applied.

## 3 Results

### 3.1 Study population and baseline characteristics

**Figure 1:**
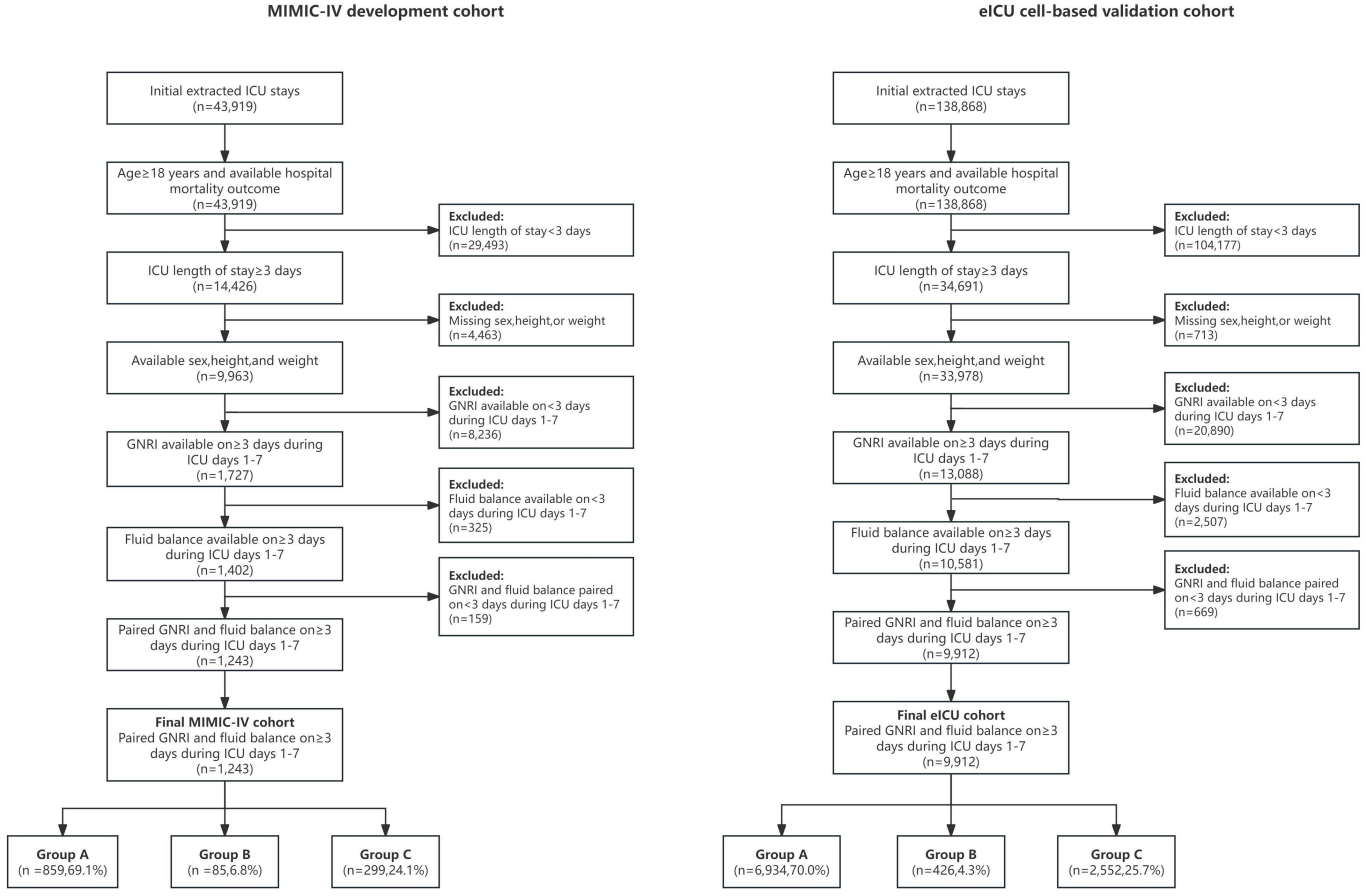
Flowchart of patient selection in the MIMIC-IV development cohort and eICU reproducibility cohort. GNRI, Geriatric Nutritional Risk Index; ICU, intensive care unit.

In MIMIC-IV, 43,919 adult ICU stays were initially extracted. After applying the predefined inclusion and exclusion criteria, 1,243 patients were included in the development cohort. In eICU, 138,868 adult ICU stays were screened, and 9,912 patients were included in the cell-based reproducibility cohort. The major exclusions were ICU length of stay less than 3 days, unavailable sex, height, or weight, and insufficient paired nutritional risk-fluid balance observations. The cohort selection flow is shown in Figure 1. Included and excluded patients differed in illness severity, length of stay, nutritional-fluid baseline variables, organ support use, and mortality, indicating that the findings apply to patients with sufficient repeated nutritional-fluid measurements rather than to all ICU admissions (Supplementary Table S2).

Baseline characteristics stratified by the three joint trajectory groups are shown separately for the MIMIC-IV development cohort and the eICU reproducibility cohort in Tables 1A, 1B, respectively. In both cohorts, Group B, characterized by initially severe nutritional risk with high fluid load and rapid decline, had the lowest baseline GNRI and the highest nutritional risk score. Group B also showed greater illness severity, reflected by higher SOFA score in MIMIC-IV and higher APACHE score in eICU, and had higher use of norepinephrine and higher crude mortality than the other trajectory groups.

**Table 1A:** Baseline characteristics of the MIMIC-IV development cohort stratified by joint trajectory group.

Variable	Total	Group A	Group B	Group C	*P* value
Demographics					
Age, years	60.0 [48.0; 71.0]	59.0 [48.0; 71.0]	59.0 [48.0; 66.0]	61.0 [50.0; 73.0]	0.362
Male sex	757 (60.9%)	528 (61.5%)	45 (52.9%)	184 (61.5%)	0.297
Body mass index, kg/m^2^	28.4 [24.5; 33.4]	28.3 [24.6; 33.4]	29.9 [24.1; 34.4]	28.5 [24.5; 32.8]	0.707
Body weight, kg	82.9 [69.8; 98.6]	82.0 [69.2; 97.8]	82.0 [68.0; 100.0]	85.0 [70.4; 100.0]	0.541
Nutritional status					
Baseline GNRI	84.9 [77.4; 90.8]	81.9 [75.9; 87.1]	78.9 [71.9; 84.8]	93.8 [88.7; 98.3]	< 0.001
Baseline nutritional risk score (98-GNRI)	13.1 [7.2; 20.6]	16.1 [10.9; 22.1]	19.1 [13.2; 26.1]	4.18 [-0.28; 9.25]	< 0.001
Baseline albumin, g/dL	2.90 [2.50; 3.30]	2.70 [2.35; 3.10]	2.50 [2.08; 2.90]	3.50 [3.20; 3.83]	< 0.001
Physiology and laboratory data					
Heart rate, beats/min	90.5 [78.3; 104.4]	91.3 [78.9; 104.7]	93.9 [79.8; 104.4]	88.1 [76.9; 103.1]	0.234
Respiratory rate, breaths/min	20.5 [17.9; 24.0]	20.9 [18.0; 24.3]	19.3 [16.8; 22.1]	20.1 [17.5; 23.6]	0.002
Mean blood pressure, mmHg	76.6 [71.0; 83.4]	76.6 [70.9; 82.8]	73.7 [70.1; 79.6]	77.2 [71.5; 85.9]	0.007
White blood cell count	12.1 [8.6; 17.2]	12.2 [8.6; 17.5]	12.2 [8.3; 17.1]	12.1 [8.6; 16.8]	0.987
Creatinine, mg/dL	1.17 [0.80; 2.08]	1.20 [0.80; 2.13]	1.13 [0.80; 1.90]	1.10 [0.84; 1.79]	0.475
Blood urea nitrogen, mg/dL	22.2 [14.7; 39.7]	23.2 [14.7; 41.5]	21.3 [13.8; 36.7]	21.0 [14.5; 35.5]	0.180
Lactate, mmol/L	2.17 [1.43; 3.61]	2.15 [1.43; 3.57]	2.33 [1.67; 4.40]	2.18 [1.43; 3.62]	0.268
Fluid status					
Day-1 fluid balance, mL/kg/day	31.8 [7.1; 80.1]	38.2 [9.3; 86.2]	41.2 [14.6; 105.2]	20.5 [1.1; 52.5]	< 0.001
Fluid overload > 10%	233 (18.7%)	176 (20.5%)	24 (28.2%)	33 (11.0%)	< 0.001
Comorbidities					
Heart failure	318 (25.6%)	219 (25.5%)	13 (15.3%)	86 (28.8%)	0.042
Atrial fibrillation	363 (29.2%)	247 (28.8%)	30 (35.3%)	86 (28.8%)	0.441
Hypertension	667 (53.7%)	457 (53.2%)	37 (43.5%)	173 (57.9%)	0.058
Diabetes mellitus	311 (25.0%)	226 (26.3%)	15 (17.6%)	70 (23.4%)	0.162
Chronic kidney disease	199 (16.0%)	153 (17.8%)	12 (14.1%)	34 (11.4%)	0.029
Sepsis	1,143 (92.0%)	791 (92.1%)	80 (94.1%)	272 (91.0%)	0.622
Treatments within 24 h					
Mechanical ventilation	969 (78.0%)	681 (79.3%)	64 (75.3%)	224 (74.9%)	0.243
Renal replacement therapy	141 (11.3%)	95 (11.1%)	8 (9.4%)	38 (12.7%)	0.625
Furosemide use	333 (26.8%)	233 (27.1%)	26 (30.6%)	74 (24.7%)	0.520
Spironolactone use	17 (1.4%)	12 (1.4%)	2 (2.4%)	3 (1.0%)	0.634
Norepinephrine use	563 (45.3%)	411 (47.8%)	46 (54.1%)	106 (35.5%)	< 0.001
Severity and outcomes					
SOFA score	8.00 [5.00; 11.00]	9.00 [5.00; 11.00]	10.0 [8.0; 13.0]	7.00 [4.00; 10.00]	< 0.001
Charlson comorbidity index	4.00 [2.50; 6.00]	4.00 [2.00; 7.00]	4.00 [3.00; 6.00]	4.00 [3.00; 6.00]	0.972
Hospital mortality	350 (28.2%)	228 (26.5%)	34 (40.0%)	88 (29.4%)	0.027
30-day mortality/30-day in-hospital mortality	357 (28.7%)	233 (27.1%)	36 (42.4%)	88 (29.4%)	0.012

Values are median [interquartile range] or n (%). Group A indicates persistent moderate-to-high nutritional risk with gradual deresuscitation; Group B indicates initially severe nutritional risk with high fluid load and rapid decline; Group C indicates worsening nutritional risk with mild-to-moderate deresuscitation. GNRI, Geriatric Nutritional Risk Index; RRT, renal replacement therapy; SOFA, Sequential Organ Failure Assessment.

**Table 1B:** Baseline characteristics of the eICU reproducibility cohort stratified by joint trajectory group.

Variable	Total	Group A	Group B	Group C	*P* value
Demographics					
Age, years	65.0 [53.0; 75.0]	65.0 [53.0; 75.0]	61.0 [51.0; 72.8]	65.0 [53.0; 75.0]	0.003
Male sex	5,608 (56.6%)	3,859 (55.7%)	201 (47.2%)	1,548 (60.7%)	< 0.001
Body mass index, kg/m^2^	27.8 [23.6; 33.4]	27.8 [23.5; 33.5]	27.1 [22.7; 32.6]	28.1 [24.1; 33.3]	0.020
Body weight, kg	81.3 [67.3; 98.6]	80.8 [66.7; 98.3]	76.2 [62.5; 94.7]	83.0 [69.1; 100.0]	< 0.001
Nutritional status					
Baseline GNRI	81.8 [74.5; 87.9]	78.9 [73.0; 84.9]	72.4 [65.5; 78.2]	89.3 [83.4; 94.6]	< 0.001
Baseline nutritional risk score (98-GNRI)	16.2 [10.1; 23.5]	19.1 [13.1; 25.0]	25.6 [19.8; 32.5]	8.65 [3.36; 14.61]	< 0.001
Baseline albumin, g/dL	2.70 [2.20; 3.20]	2.60 [2.13; 3.00]	2.10 [1.70; 2.50]	3.20 [2.80; 3.60]	< 0.001
Physiology and laboratory data					
Heart rate, beats/min	89.5 [77.8; 102.5]	89.9 [78.2; 102.7]	93.6 [80.8; 106.7]	87.8 [76.1; 101.2]	< 0.001
Respiratory rate, breaths/min	19.8 [16.8; 23.7]	19.9 [16.8; 23.7]	20.5 [17.0; 24.3]	19.5 [16.7; 23.5]	0.023
Mean blood pressure, mmHg	77.2 [70.3; 86.4]	76.7 [70.1; 85.5]	72.4 [67.6; 78.0]	79.6 [72.0; 89.6]	< 0.001
White blood cell count	12.0 [8.3; 16.9]	11.7 [8.2; 16.9]	13.0 [8.1; 19.3]	12.3 [8.9; 16.7]	0.002
Creatinine, mg/dL	1.23 [0.81; 2.12]	1.23 [0.80; 2.20]	1.58 [0.94; 2.73]	1.19 [0.82; 1.89]	< 0.001
Blood urea nitrogen, mg/dL	24.4 [15.0; 42.0]	25.0 [15.0; 43.0]	30.0 [18.3; 49.7]	21.7 [14.0; 36.0]	< 0.001
Lactate, mmol/L	2.02 [1.30; 3.30]	1.98 [1.27; 3.23]	2.55 [1.50; 4.35]	2.20 [1.40; 3.37]	< 0.001
Fluid status					
Day-1 fluid balance, mL/kg/day	−0.94 [−15.24; 23.57]	−0.76 [−15.31; 24.02]	2.90 [−10.70; 31.40]	−1.70 [−16.06; 21.56]	< 0.001
Fluid overload >10%	191 (1.9%)	137 (2.0%)	18 (4.2%)	36 (1.4%)	< 0.001
Comorbidities					
Heart failure	2,206 (22.3%)	1,605 (23.1%)	88 (20.7%)	513 (20.1%)	0.005
Atrial fibrillation	1,829 (18.5%)	1,267 (18.3%)	56 (13.1%)	506 (19.8%)	0.003
Hypertension	5,195 (52.4%)	3,613 (52.1%)	190 (44.6%)	1,392 (54.5%)	< 0.001
Diabetes mellitus	3,106 (31.3%)	2,216 (32.0%)	101 (23.7%)	789 (30.9%)	0.002
Chronic kidney disease	3,245 (32.7%)	2,270 (32.7%)	180 (42.3%)	795 (31.2%)	< 0.001
Sepsis	3,488 (35.2%)	2,612 (37.7%)	234 (54.9%)	642 (25.2%)	< 0.001
Treatments within 24 h					
Mechanical ventilation	5,911 (59.6%)	4,118 (59.4%)	272 (63.8%)	1,521 (59.6%)	0.190
Renal replacement therapy	768 (7.7%)	565 (8.1%)	48 (11.3%)	155 (6.1%)	< 0.001
Furosemide use	1,827 (18.4%)	1,298 (18.7%)	78 (18.3%)	451 (17.7%)	0.506
Spironolactone use	80 (0.8%)	58 (0.8%)	4 (0.9%)	18 (0.7%)	0.780
Norepinephrine use	3,442 (34.7%)	2,414 (34.8%)	228 (53.5%)	800 (31.3%)	< 0.001
Severity and outcomes					
APACHE score	71.0 [53.0; 92.0]	72.0 [54.0; 92.0]	85.0 [67.0; 109.0]	68.0 [50.0; 90.0]	< 0.001
Hospital mortality	1,879 (19.0%)	1,218 (17.6%)	148 (34.7%)	513 (20.1%)	< 0.001
30-day mortality/30-day in-hospital mortality	1,788 (18.0%)	1,152 (16.6%)	141 (33.1%)	495 (19.4%)	< 0.001

Values are median [interquartile range] or n (%). Fluid balance in the eICU reproducibility cohort was derived from cell-level input-output records. APACHE, Acute Physiology and Chronic Health Evaluation; GNRI, Geriatric Nutritional Risk Index; RRT, renal replacement therapy.

### 3.2 Joint trajectory phenotypes

**Figure 2:**
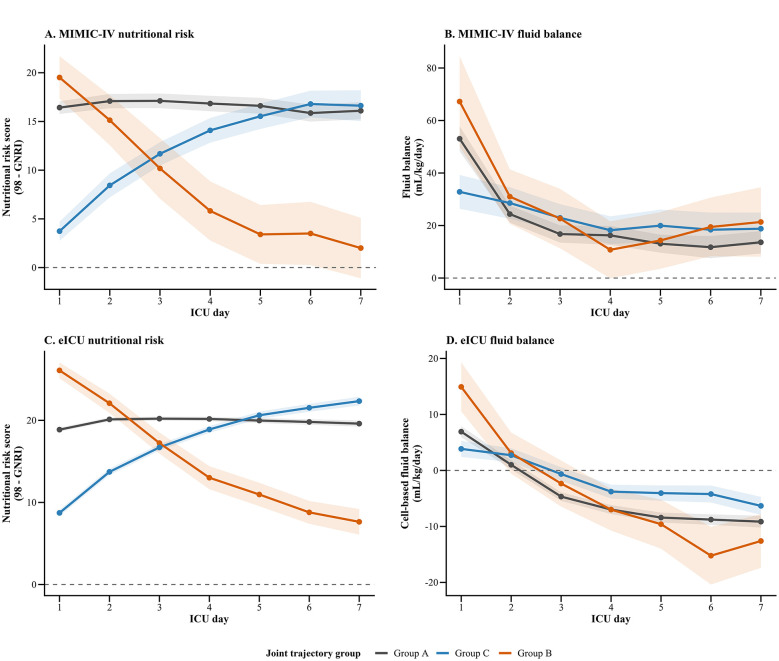
Joint trajectories of nutritional risk and fluid balance in MIMIC-IV and eICU. **(A)** and **(B)** show nutritional risk and fluid balance trajectories in MIMIC-IV; **(C)** and **(D)** show corresponding trajectories in eICU using cell-based fluid balance. Nutritional risk was defined as 98 minus GNRI. Fluid balance was standardized by baseline body weight.

The three-class joint trajectory model identified clinically interpretable patterns of nutritional risk and fluid balance in MIMIC-IV and reproduced analogous patterns in eICU (Table 2 and Figure 2). Group A was characterized by persistent moderate-to-high nutritional risk with gradual deresuscitation. Group B represented an initially severe nutritional risk phenotype with high early fluid load and rapid decline, and it was the smallest but highest-risk group. Group C showed a pattern of worsening nutritional risk with mild-to-moderate deresuscitation. In MIMIC-IV, the proportions of Group A, Group B, and Group C were 69.1%, 6.8%, and 24.1%, respectively. In eICU, the corresponding proportions were 70.0%, 4.3%, and 25.7%. AvePP values were above 0.79 in MIMIC-IV and above 0.81 in eICU, supporting acceptable average posterior classification, although OCC values for the dominant Group A were below 5 and were therefore interpreted cautiously (Supplementary Table S3). Four-class trajectory models are summarized in Supplementary Table S4 and Supplementary Figure S4.

**Table 2:** Three-class joint trajectory model classification and clinical summary.

Cohort	Group	*n* (%)	AvePP	OCC	Hospital mortality	30-day mortality	GNRI median	Severity median
MIMIC-IV	Group A	859 (69.1%)	0.840	2.35	26.5%	27.1%	81.9	9
MIMIC-IV	Group B	85 (6.8%)	0.887	106.53	40.0%	42.4%	78.9	10
MIMIC-IV	Group C	299 (24.1%)	0.792	11.99	29.4%	29.4%	93.8	7
eICU	Group A	6,934 (70.0%)	0.856	2.54	17.6%	16.6%	78.9	72
eICU	Group B	426 (4.3%)	0.905	213.23	34.7%	33.1%	72.4	85
eICU	Group C	2,552 (25.7%)	0.815	12.72	20.1%	19.4%	89.3	68

In MIMIC-IV, the severity median refers to SOFA score; in eICU, the severity median refers to APACHE score. AvePP, average posterior probability; OCC, odds of correct classification; GNRI, Geriatric Nutritional Risk Index.

### 3.3 Mortality risk across joint trajectory groups

In the development cohort, hospital mortality was 26.5% in Group A, 40.0% in Group B, and 29.4% in Group C. Thirty-day mortality was 27.1%, 42.4%, and 29.4%, respectively, and 90-day mortality was 33.1%, 49.4%, and 37.8%, respectively. In eICU, hospital mortality was 17.6% in Group A, 34.7% in Group B, and 20.1% in Group C, while 30-day in-hospital mortality was 16.6%, 33.1%, and 19.4%, respectively (Table 2).

**Figure 3:**
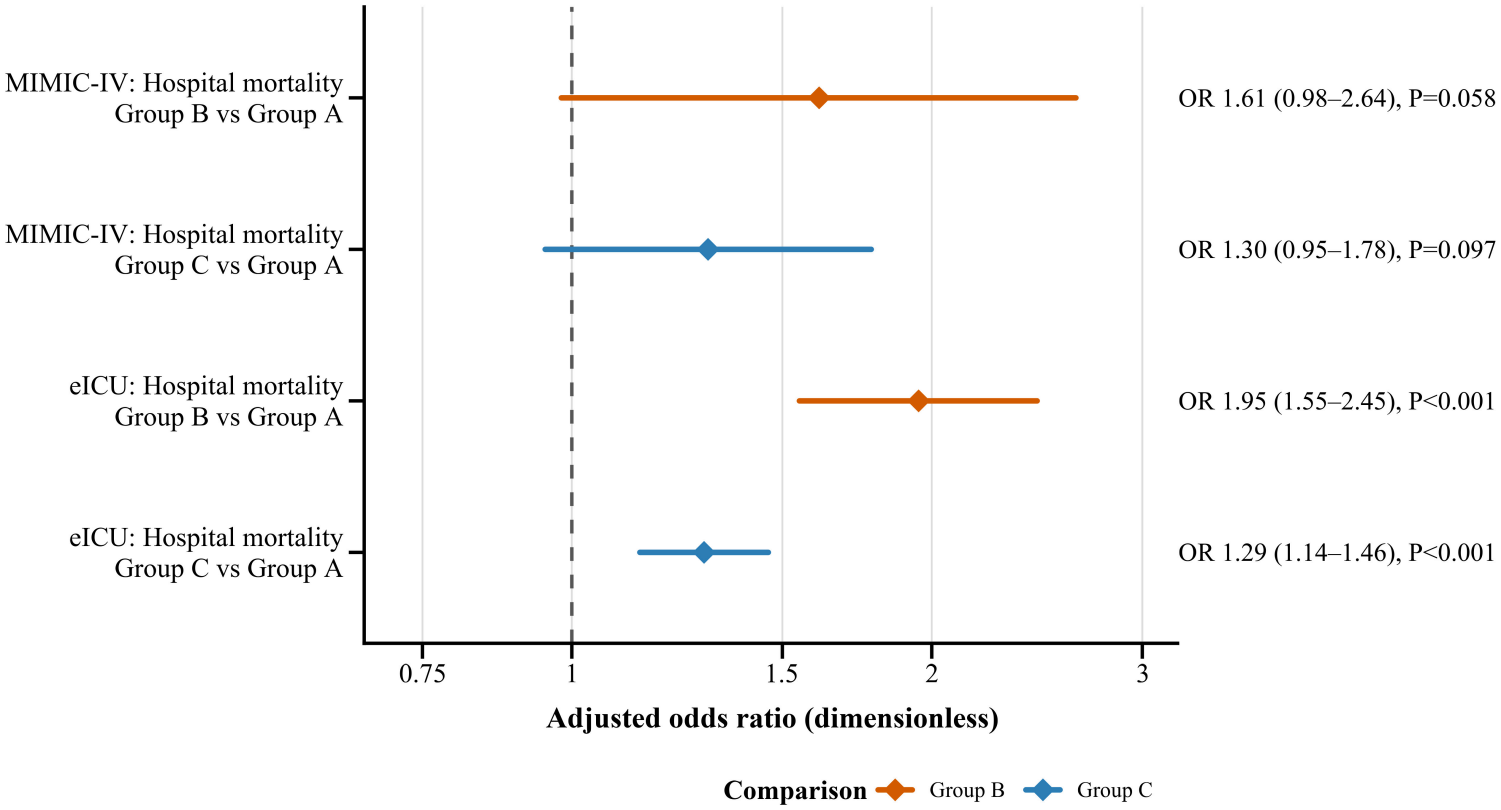
Fully adjusted associations between joint trajectory groups and hospital mortality. Group A served as the reference group. Estimates are adjusted odds ratios from Model 4.

In fully adjusted logistic models for hospital mortality, Group B showed a consistent high-risk direction in MIMIC-IV (OR 1.61, 95% CI 0.98–2.64; *P* = 0.058) and a significant association in eICU (OR 1.95, 95% CI 1.55–2.45; *P* < 0.001). Group C was associated with increased hospital mortality in eICU (OR 1.29, 95% CI 1.14–1.46; *P* < 0.001), while the corresponding MIMIC-IV estimate was directionally similar but not statistically significant (OR 1.30, 95% CI 0.95–1.78; *P* = 0.097). These associations are shown in Figure 3 and summarized in Table 3.

**Table 3:** Fully adjusted associations between joint trajectory groups and clinical outcomes.

Cohort	Outcome	Comparison	Measure	Estimate (95% CI)	*P* value	*n*	Events
MIMIC-IV	Hospital mortality	Group B vs. Group A	OR	1.61 (0.98–2.64)	0.058	1,243	350
MIMIC-IV	Hospital mortality	Group C vs. Group A	OR	1.30 (0.95–1.78)	0.097	1,243	350
MIMIC-IV	30-day mortality	Group B vs. Group A	HR	1.59 (1.12–2.28)	0.010	1,243	357
MIMIC-IV	30-day mortality	Group C vs. Group A	HR	1.28 (1.00–1.65)	0.051	1,243	357
MIMIC-IV	90-day mortality	Group B vs. Group A	HR	1.60 (1.15–2.22)	0.005	1,243	439
MIMIC-IV	90-day mortality	Group C vs. Group A	HR	1.37 (1.10–1.72)	0.006	1,243	439
eICU	Hospital mortality	Group B vs. Group A	OR	1.95 (1.55–2.45)	< 0.001	9,090	1,760
eICU	Hospital mortality	Group C vs. Group A	OR	1.29 (1.14–1.46)	< 0.001	9,090	1,760
eICU	30-day in-hospital mortality	Group B vs. Group A	OR	1.93 (1.53–2.44)	< 0.001	9,090	1,674
eICU	30-day in-hospital mortality	Group C vs. Group A	OR	1.32 (1.16–1.50)	< 0.001	9,090	1,674

**Figure 4:**
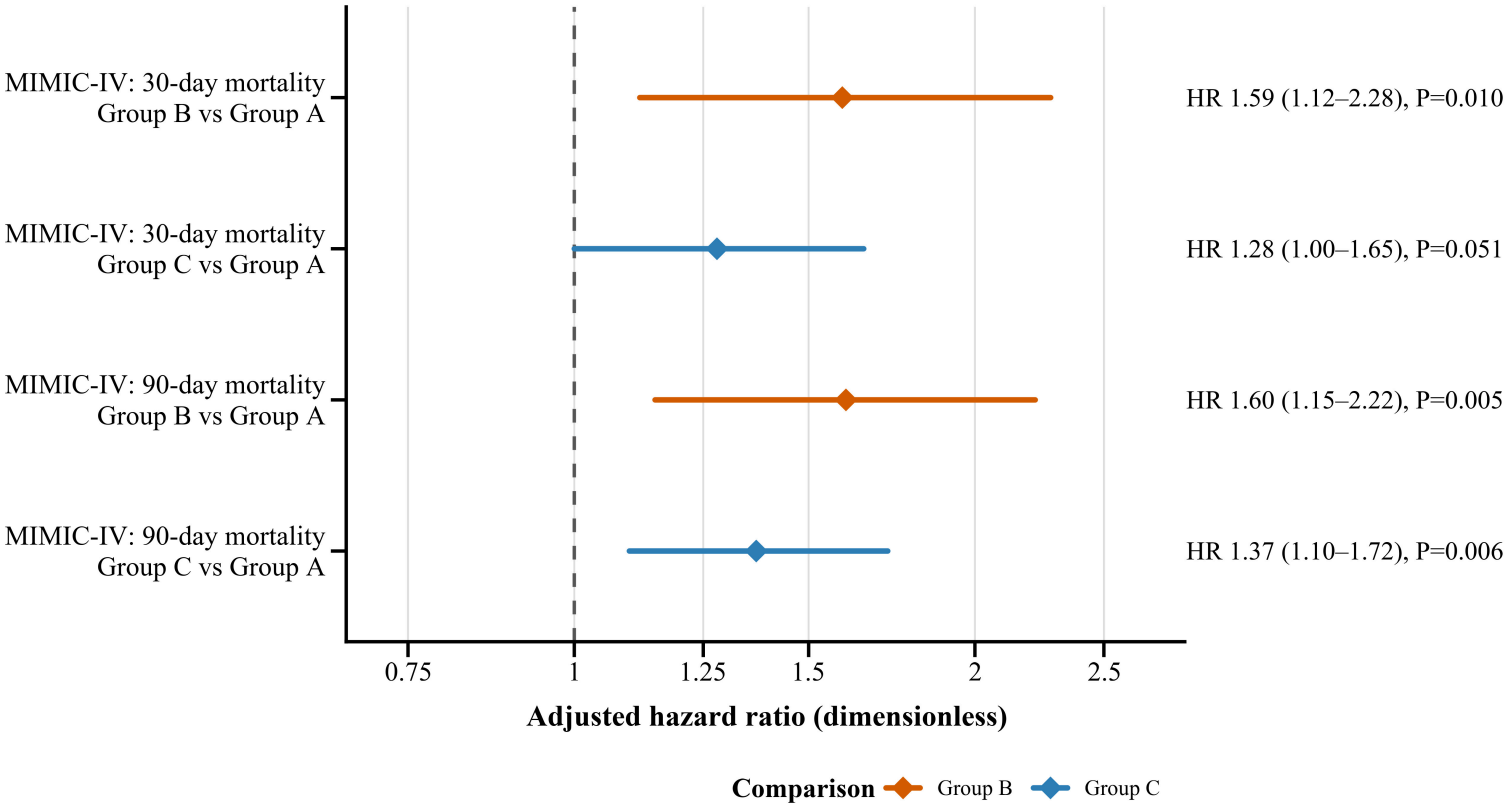
Fully adjusted associations between joint trajectory groups and 30-day and 90-day mortality in MIMIC-IV. Group A served as the reference group. Estimates are adjusted hazard ratios from Model 4.

In MIMIC-IV, Group B was significantly associated with higher 30-day mortality (HR 1.59, 95% CI 1.12–2.28; *P* = 0.010) and 90-day mortality (HR 1.60, 95% CI 1.15–2.22; *P* = 0.005). Group C was associated with higher 90-day mortality (HR 1.37, 95% CI 1.10–1.72; *P* = 0.006), while the association with 30-day mortality approached statistical significance (HR 1.28, 95% CI 1.00–1.65; *P* = 0.051). The MIMIC-IV Cox model results are displayed in Figure 4 and Table 3.

### 3.4 Survival analysis

**Figure 5:**
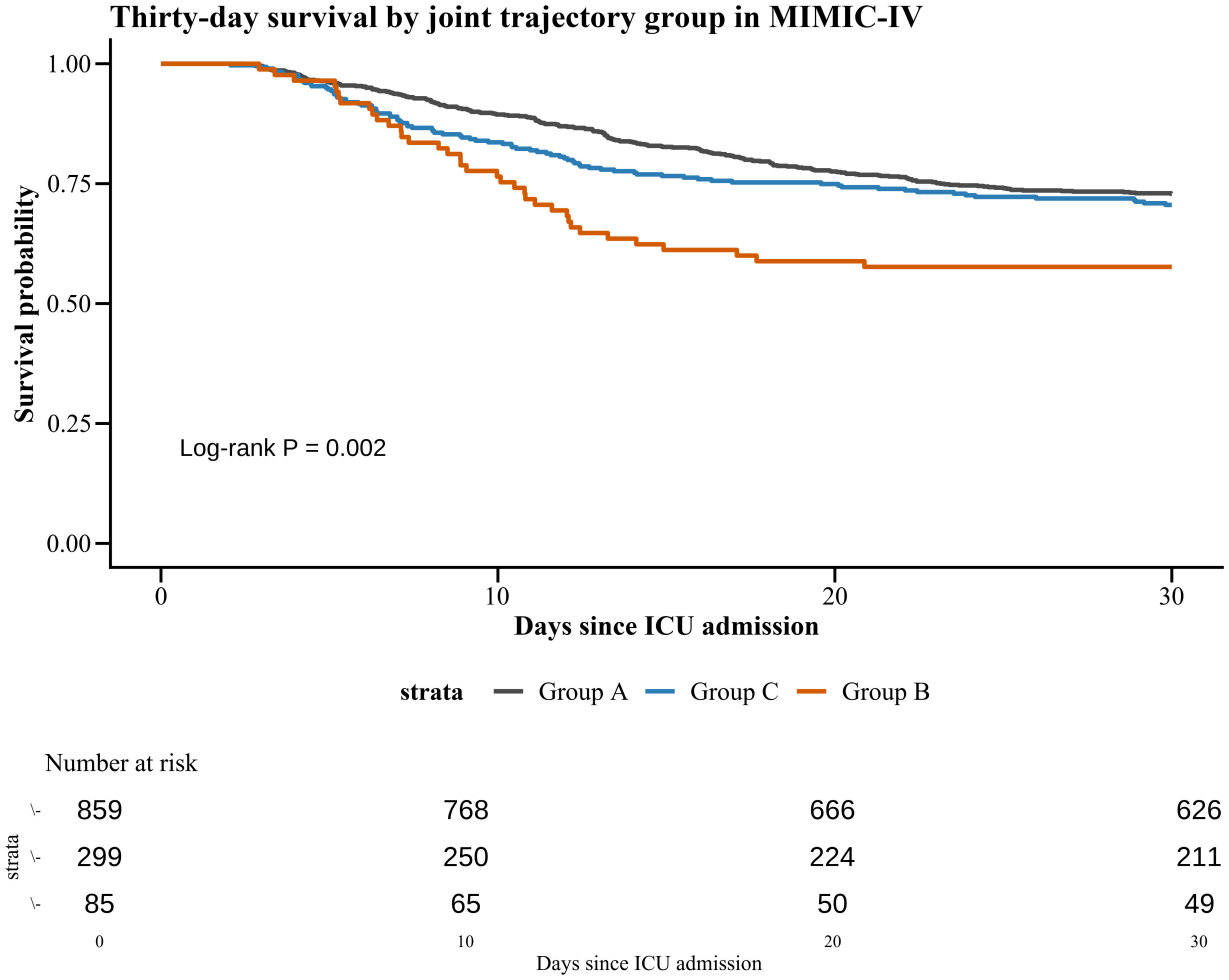
Kaplan-Meier curves for 30-day mortality in MIMIC-IV according to joint trajectory group. The log-rank test was used to compare survival curves.

Kaplan-Meier curves demonstrated significant differences in 30-day survival across the three joint trajectory groups in MIMIC-IV (log-rank *P* = 0.002; Figure 5). Group B had the lowest 30-day survival, consistent with its severe early nutritional risk and high fluid load. Ninety-day survival differences in MIMIC-IV were also significant (log-rank *P* = 0.001; Supplementary Figure S1). In eICU, 30-day in-hospital survival curves also differed across trajectory groups (log-rank *P* < 0.001; Supplementary Figure S2), supporting reproducibility of prognostic separation across databases.

### 3.5 Subgroup analyses

**Figure 6:**
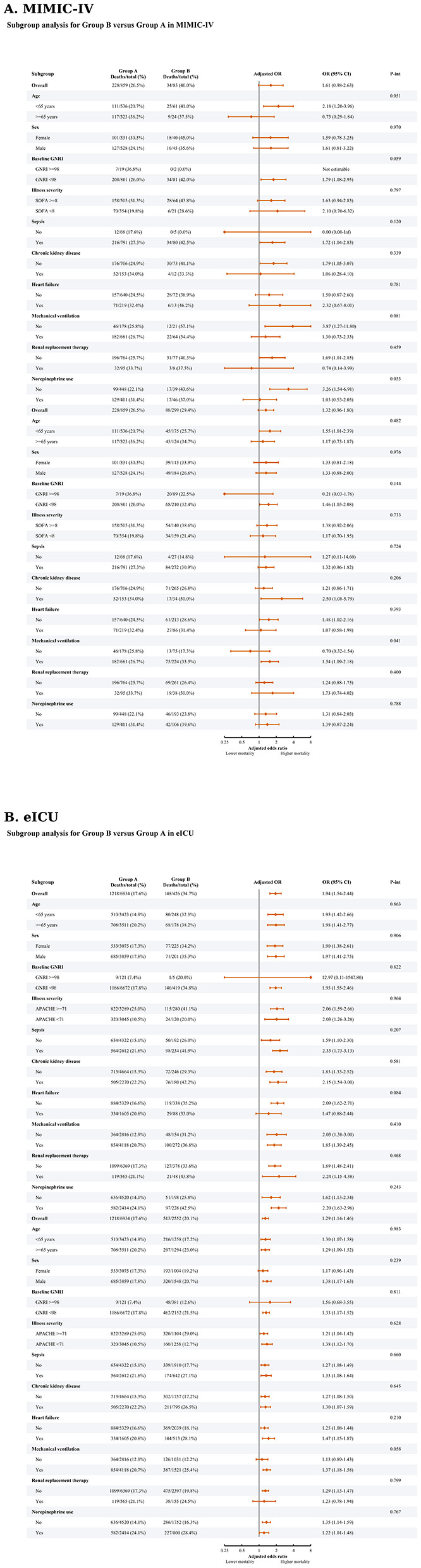
Prespecified subgroup analyses for Group B vs. Group A. **(A)** shows MIMIC-IV; **(B)** shows eICU. Estimates are adjusted odds ratios for hospital mortality. *P*-int indicates *P* for interaction.

Prespecified subgroup analyses focused on Group B vs. Group A, given that Group B represented the highest-risk phenotype. In eICU, the association between Group B and hospital mortality was directionally consistent across age, sex, baseline GNRI, severity, sepsis, chronic kidney disease, heart failure, mechanical ventilation, renal replacement therapy, and norepinephrine use strata, with no strong evidence of interaction (Figure 6B). In MIMIC-IV, estimates were directionally consistent in most subgroups, although precision was limited by the small number of patients in Group B; interaction tests were not statistically significant, with several borderline patterns likely reflecting small subgroup sizes (Figure 6A). Subgroup analyses were exploratory and were not adjusted for multiple testing. Subgroup analyses for Group C vs. Group A are presented in Supplementary Figure S5 and Supplementary Table S5.

### 3.6 Supplementary, landmark, and incremental prediction analyses

Restricted cubic spline analyses showed that mean daily fluid balance was associated with hospital mortality in both MIMIC-IV and eICU, with evidence of non-linearity (Supplementary Figure S3). Baseline nutritional risk showed a significant overall association in eICU but not in MIMIC-IV after full covariate adjustment. Candidate model-selection diagnostics, paired-observation distributions, and posterior classification diagnostics are provided in Supplementary Table S3. The four-class models identified smaller high-risk phenotypes, but because several subgroup proportions were small and clinical interpretability was less stable, the three-class model was selected as the primary model based on parsimony, reproducibility, and clinical interpretability (Supplementary Table S4 and Supplementary Figure S4).

Landmark analyses evaluated exposure-window-related and immortal time bias. In the strict day-7 landmark analysis, the eICU associations remained robust, whereas MIMIC-IV estimates were directionally consistent but imprecise (Supplementary Table S6 and Supplementary Figure S6). Short-window trajectories reconstructed from ICU days 1–3 yielded analogous phenotypes. The day-3 high-risk phenotype remained significantly associated with post-day-3 hospital mortality and post-day-3 30-day in-hospital mortality in eICU, whereas MIMIC-IV estimates were directionally above 1.0 but not statistically significant because the high-risk short-window class was small (Supplementary Tables S7, S8 and Supplementary Figures S7, S8).

Extended adjustment analyses evaluated construct overlap between GNRI-derived nutritional risk and fluid balance. After additional adjustment for baseline GNRI, baseline albumin, day-1 fluid balance, and cumulative fluid balance during ICU days 1–3, Group B remained associated with MIMIC-IV 30-day and 90-day mortality and with eICU hospital and 30-day in-hospital mortality. These findings indicated that the joint trajectory signal was not fully explained by baseline GNRI, hypoalbuminemia, or early fluid balance alone (Supplementary Table S9 and Supplementary Figure S9).

Incremental prediction analyses were performed in complete-case prediction datasets (MIMIC-IV, *n* = 1,185; eICU, *n* = 8,730). Adding joint trajectory information to baseline GNRI and early fluid balance produced modest changes in cross-validated discrimination and Brier score. Calibration curves showed broadly acceptable calibration, and decision curve analysis indicated similar but slightly higher net benefit for the full model across clinically relevant threshold ranges (Supplementary Tables S10, S11 and Supplementary Figures S10, S12). These results support modest incremental prognostic information from the joint trajectory phenotype, while indicating that its main value lies in clinically interpretable dynamic phenotyping rather than large standalone prediction gains. Schoenfeld residual tests indicated potential non-proportionality in the MIMIC-IV 30-day Cox model and in global tests for the MIMIC-IV 90-day model, whereas the 90-day trajectory-group term itself did not show evidence of non-proportionality; Cox-based estimates were therefore interpreted cautiously and considered together with fixed-time, landmark, and sensitivity analyses (Supplementary Table S12). In a high-confidence posterior classification sensitivity analysis restricted to patients with posterior probability >= 0.70, the high-risk Group B phenotype remained consistently associated with mortality, supporting the robustness of the modal class assignment approach while not replacing formal three-step or probability-weighted methods (Supplementary Table S13).

## 4 Discussion

This dual-database study identified three reproducible joint trajectories of nutritional risk and fluid balance during the first 7 ICU days. The highest-risk phenotype, Group B, combined initially severe nutritional risk with high early fluid load and rapid decline. This uncommon phenotype carried the highest hospital and short-term mortality in both the MIMIC-IV development cohort and the eICU reproducibility cohort. In fully adjusted models, Group B was associated with increased 30-day and 90-day mortality in MIMIC-IV and with significantly higher hospital mortality and 30-day in-hospital mortality in eICU. Group C, characterized by worsening nutritional risk with mild-to-moderate deresuscitation, showed a more moderate but consistent risk signal, particularly for 90-day mortality in MIMIC-IV and hospital mortality in eICU. These findings indicate that the joint temporal pattern of nutritional risk and fluid balance provides clinically meaningful prognostic information and modest incremental value beyond static baseline assessment.

Group B was the most clinically relevant phenotype. The rapid decline in fluid balance after an early high fluid load resembles transition from resuscitation to deresuscitation, but the accompanying severe nutritional risk distinguishes this phenotype from a simple recovery pattern. Group B likely represents a high-acuity state in which inflammation, capillary leak, hemodynamic instability, and early resuscitation requirements coexist with marked nutritional vulnerability. The subsequent decline in fluid balance may reflect active fluid removal, diuretic exposure, renal replacement therapy, spontaneous diuresis after shock reversal, or survival long enough to enter a deresuscitation phase. The excess mortality observed in Group B therefore reflects the prognostic meaning of the combined phenotype, not evidence that rapid fluid reduction itself is harmful. This distinction is central because fluid therapy is time-dependent, and the same numerical fluid balance may carry different implications during resuscitation, stabilization, and recovery.

The biological plausibility of this high-risk phenotype is supported by established mechanisms of critical illness. Malnutrition and nutritional risk in ICU patients have been associated with adverse clinical outcomes, including mortality, infectious complications, and prolonged organ support (16). Acute critical illness is also accompanied by accelerated skeletal muscle wasting, increased protein catabolism, and impaired nutritional reserve, changes that can emerge within the first week of ICU admission (17). At the same time, serum albumin and body weight, the two components used to calculate GNRI, are highly sensitive to inflammation, capillary leakage, dilution, edema, and fluid removal. Visceral proteins such as albumin and prealbumin are no longer considered reliable stand-alone indicators of nutritional status in acute inflammatory states, and hypoalbuminemia is increasingly recognized as a marker of inflammatory burden, vascular permeability, and disease severity rather than a pure marker of nutrient intake (18, 19). Defining nutritional risk as 98 minus daily GNRI and modeling its trajectory preserved the prognostic signal of GNRI while recognizing that its components are dynamically modified by the critical illness-fluid interaction. Accordingly, the GNRI-derived trajectory is interpreted as an inflammatory-nutritional-fluid risk signal rather than as a pure marker of nutritional depletion.

These findings are also consistent with and extend prior evidence regarding fluid balance. Positive cumulative fluid balance and fluid overload have repeatedly been associated with increased mortality in adult critical care populations, including patients with acute respiratory distress syndrome and other high-acuity ICU conditions (6–8). Conservative fluid management or deresuscitation after the initial resuscitation phase has been associated with improved fluid-related outcomes in sepsis and acute respiratory distress syndrome (7). Contemporary sepsis guidelines emphasize dynamic assessment to guide resuscitation and recognize crystalloids as first-line resuscitation fluids, while uncertainty remains regarding restrictive vs. liberal fluid strategies after early resuscitation (20). Phase-based fluid management frameworks further frame fluid therapy as a time-dependent intervention involving resuscitation, optimization, stabilization, and evacuation phases (21). Fluid overload has also shown a dose-response association with in-hospital mortality in critically ill patients (22). In this context, fluid balance requires longitudinal interpretation rather than isolated assessment, and its prognostic meaning varies according to concurrent nutritional risk.

Compared with previous trajectory-based studies, the present work adds several methodological and clinical contributions. Prior fluid balance trajectory analyses have demonstrated that latent patterns of fluid accumulation and removal are associated with outcomes in septic shock and in patients receiving renal replacement therapy (11, 12). A recent study in Frontiers in Nutrition reported that fluid balance trajectories were associated with prognosis among older malnourished ICU patients, but it focused on a single fluid-balance domain, restricted the population to older patients with GNRI-defined malnutrition, and did not include an independent multicenter reproducibility cohort (13). Our study extends this line of research by evaluating adult critically ill patients irrespective of age or baseline malnutrition category, jointly modeling nutritional risk and fluid balance, and evaluating the reproducibility of trajectory patterns and prognostic associations in a large multicenter eICU cohort. The three-class solution was selected not solely because of statistical indices, but because it provided a reproducible and parsimonious clinical structure across databases. The four-class models identified smaller high-risk phenotypes, but the three-class model offered a better balance between interpretability, stability, and external reproducibility.

The reproducibility of the three joint trajectory groups across MIMIC-IV and eICU strengthens the credibility of the findings. Group A represented a large reference phenotype with persistent moderate-to-high nutritional risk and gradual deresuscitation. Group C represented patients whose nutritional risk worsened over time despite mild-to-moderate negative fluid balance, consistent with delayed or progressive nutritional deterioration. Group B represented the highest-risk nutritional-fluid phenotype. Importantly, the database-specific severity adjustment differed between cohorts: MIMIC-IV models used SOFA and Charlson scores, whereas eICU models used APACHE score because SOFA and Charlson were unavailable or completely missing in eICU. Despite these differences in covariate structure and data collection, the direction of effect was consistent across cohorts, supporting the reproducibility of the main signal.

The findings have several clinical implications. First, they support dynamic joint assessment during the early ICU course rather than reliance on static nutritional or fluid metrics alone. A single GNRI, albumin value, or cumulative fluid balance may miss patients whose risk emerges through the interaction of nutritional deterioration and fluid therapy. Second, the high-risk Group B phenotype can serve as an early warning pattern for multidisciplinary reassessment of nutritional delivery, fluid responsiveness, cumulative input from medications and nutrition formulas, diuretic or renal replacement therapy strategy, and ongoing inflammatory or hemodynamic instability. Third, the trajectory approach can distinguish patients with stable moderate risk from those with worsening nutritional risk, a group that may be overlooked when only baseline GNRI is considered. These implications represent dynamic risk phenotyping rather than treatment recommendations. The present study does not establish that altering fluid balance or nutrition support according to trajectory group improves outcomes.

Supplementary analyses clarified the robustness and interpretation of the joint trajectory approach. Kaplan-Meier curves showed separation of survival probabilities across trajectory groups, with the lowest survival in the high-risk Group B phenotype. Subgroup analyses focused on Group B vs. Group A showed directionally consistent associations across clinically relevant strata, although precision was limited in MIMIC-IV because Group B was relatively small. Restricted cubic spline analyses indicated that continuous nutritional risk and mean daily fluid balance were associated with mortality in a dose-response manner, particularly in the larger eICU cohort. Landmark and short-window analyses reduced concerns that the main findings were driven solely by exposure-window-related selection or immortal time bias, although MIMIC-IV post-landmark estimates remained imprecise. Extended adjustment analyses showed that the high-risk phenotype was not fully explained by baseline GNRI, baseline albumin, day-1 fluid balance, or early cumulative fluid balance. Incremental prediction analyses demonstrated modest additional prognostic information beyond baseline GNRI and early fluid balance, although the magnitude of improvement was limited. Proportional-hazards diagnostics indicated potential non-proportionality for the MIMIC-IV 30-day Cox model, supporting cautious interpretation of hazard ratios and emphasizing corroborating evidence from fixed-time, landmark, and sensitivity analyses. A high-confidence posterior classification sensitivity analysis further supported the robustness of the high-risk phenotype. The joint trajectory model is therefore best interpreted as a clinically interpretable dynamic phenotyping tool rather than a replacement for conventional severity scores or a standalone prediction model.

This study has several strengths. First, it used two large, publicly available ICU databases, with MIMIC-IV serving as the development cohort and eICU used to evaluate external reproducibility. Second, it modeled nutritional risk and fluid balance simultaneously using group-based multi-trajectory modeling, thereby capturing joint dynamic phenotypes rather than analyzing each domain in isolation. Third, the analysis used daily data over the first 7 ICU days and required at least three paired observations, improving the reliability of trajectory assignment. Fourth, the eICU analysis used cell-level input and output records to derive fluid balance, addressing known challenges in interpreting cumulative total fields in eICU intake-output data. Previous systematic work has emphasized that fluid balance records and body weight can be imperfect surrogates for body fluid status in critically ill adults (23). Finally, the analysis incorporated sequential multivariable adjustment, survival analysis, subgroup analyses, restricted cubic splines, four-class sensitivity models, landmark analyses, extended adjustment for construct overlap, and incremental prediction analyses.

The study has several limitations. First, the retrospective observational design precludes causal inference. The identified trajectories may reflect underlying disease severity, treatment intensity, or residual confounding by indication rather than modifiable exposure patterns. Second, patients were required to have an ICU stay of at least 3 days and at least three paired nutritional risk-fluid balance observations during the first 7 ICU days. This criterion may have excluded patients who died very early, recovered rapidly, or had incomplete documentation, creating selection bias and potential immortal time bias (24). Included-versus-excluded comparisons and strict day-7 and day-3 landmark sensitivity analyses reduced, but did not eliminate, concerns about selection and exposure-window-related bias; therefore, the findings apply to critically ill adults with sufficient repeated nutritional-fluid measurements. Third, GNRI depends on albumin and body weight, both of which are altered by inflammation and fluid status. Although extended adjustment analyses indicated that the joint trajectory signal was not fully explained by baseline GNRI, hypoalbuminemia, or early fluid balance, GNRI-derived nutritional risk represents an inflammatory-nutritional-fluid risk signal rather than a direct measure of malnutrition. A harmonized recalculation of daily GNRI using fixed baseline body weight was not feasible because consistently time-stamped daily weights were unavailable across both databases. Fourth, patients were assigned to the most likely trajectory group for outcome modeling. Although posterior probability diagnostics supported acceptable average classification quality and high-confidence posterior sensitivity analyses were consistent with the main findings, the regression models did not formally incorporate posterior classification uncertainty using a three-step or probability-weighted approach. Class-assignment uncertainty may therefore have affected standard errors and effect estimates, particularly for the small Group B. Fifth, Schoenfeld residual tests indicated potential non-proportionality in the MIMIC-IV 30-day Cox model and in global tests for the 90-day model. Cox-based estimates were interpreted together with fixed-time logistic, landmark, and posterior-sensitivity analyses. Sixth, fluid balance extraction differed between databases. MIMIC-IV used input-output event data, whereas eICU required cell-level intake and output records for the main analysis. Differences in documentation practices could have affected absolute fluid balance values, although the eICU analysis focused primarily on trajectory shape and prognostic association. Seventh, detailed nutritional exposures, including daily energy and protein targets, feeding interruptions, enteral or parenteral nutrition composition, and the fluid contribution of nutrition formulas, were not fully available. Eighth, severity adjustment was database-specific, and several candidate variables such as lactate or temperature had substantial missingness in eICU and were not included in the primary models. Finally, the high-risk Group B phenotype was relatively small, especially in MIMIC-IV; although posterior classification diagnostics and eICU reproducibility were supportive, the estimates remain imprecise.

In summary, early joint trajectories of GNRI-derived nutritional risk and fluid balance identified clinically interpretable prognostic phenotypes in selected critically ill adults with sufficient repeated measurements. The highest-risk phenotype combined severe early nutritional risk with high early fluid load and rapid decline, and this pattern was reproduced across two independent ICU databases. The prognostic meaning of nutritional risk and fluid balance is best understood dynamically and jointly, while the incremental statistical prediction gain beyond conventional severity, baseline GNRI, and early fluid balance remains modest.

## 5 Conclusion

Joint trajectories of GNRI-derived nutritional risk and fluid balance during the first 7 ICU days were associated with prognosis in selected critically ill adults with sufficient repeated measurements. A phenotype characterized by initially severe nutritional risk and high early fluid load with rapid decline was reproducibly identified across MIMIC-IV and eICU and was associated with the highest mortality risk. Dynamic joint assessment of nutrition-fluid trajectories provides a clinically interpretable risk phenotyping approach that complements, rather than replaces, conventional severity assessment.

## Data Availability

Publicly available datasets were analyzed in this study. MIMIC-IV is available at https://physionet.org/content/mimiciv/, and the eICU Collaborative Research Database is available at https://physionet.org/content/eicu-crd/. Access requires completion of the relevant PhysioNet credentialing/training and data use agreement.
