# Supplementary material for: Joint trajectories of nutritional risk and fluid balance and prognosis in critically ill adults: a dual-trajectory modeling study using MIMIC-IV and eICU
Source: Front Nutr. 2026 Jul 20;13:1881675. doi: 10.3389/fnut.2026.1881675 (PMC13429405; doi:10.3389/fnut.2026.1881675)
Supplement: Supplementary file 1 [file Data_Sheet_1.docx]

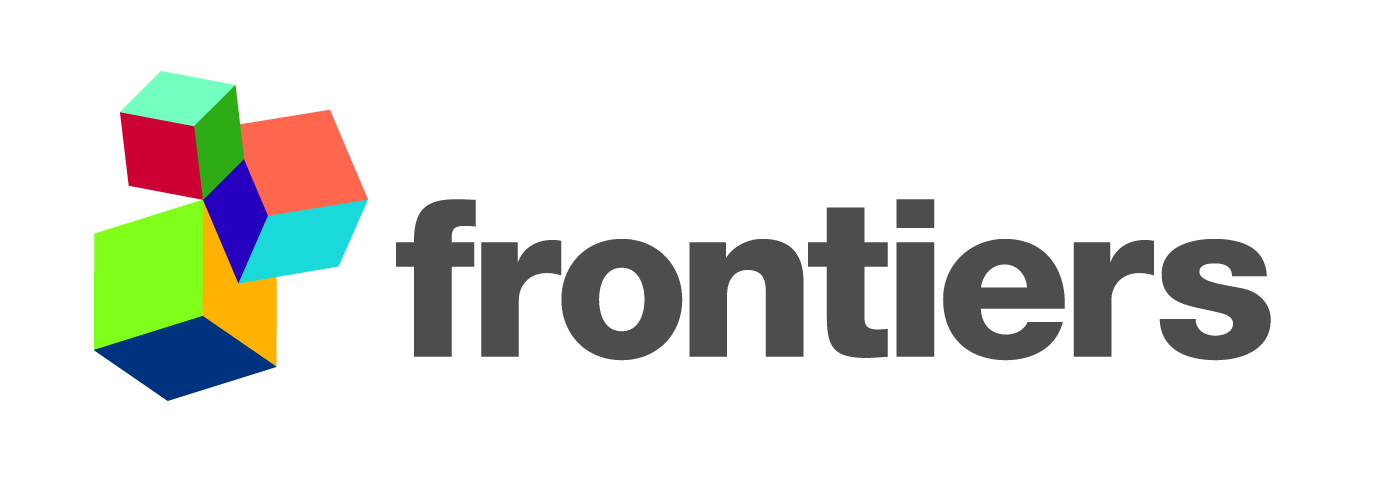


**Supplementary Material**

Joint trajectories of nutritional risk and fluid balance and prognosis in critically ill adults: a dual-trajectory modeling study using MIMIC-IV and eICU

**1 Supplementary Figures**

**Supplementary Figure S1. Kaplan-Meier curves for 90-day mortality in MIMIC-IV according to joint trajectory group.**


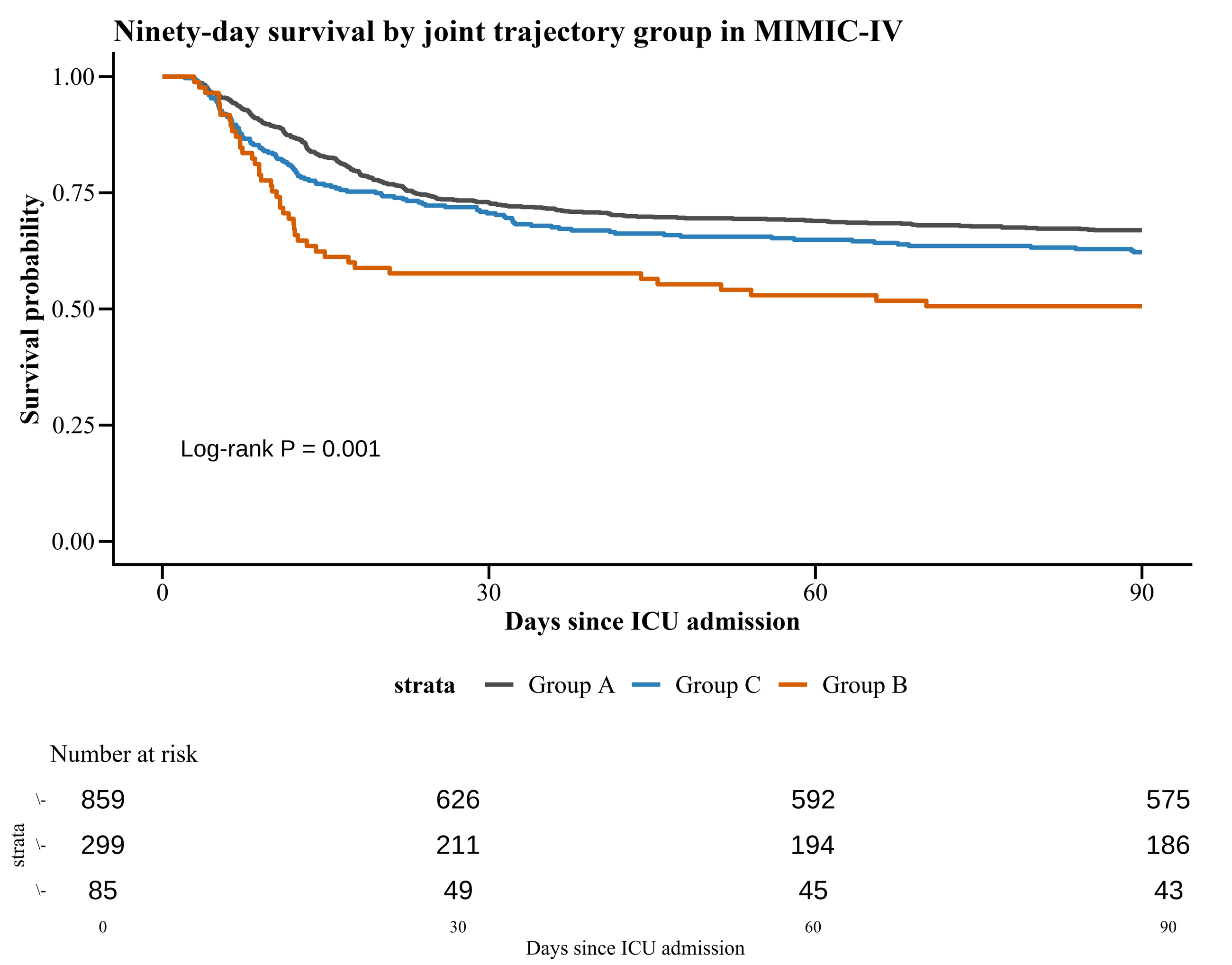


**Supplementary Figure S2. Kaplan-Meier curves for 30-day in-hospital mortality in eICU according to joint trajectory group.**


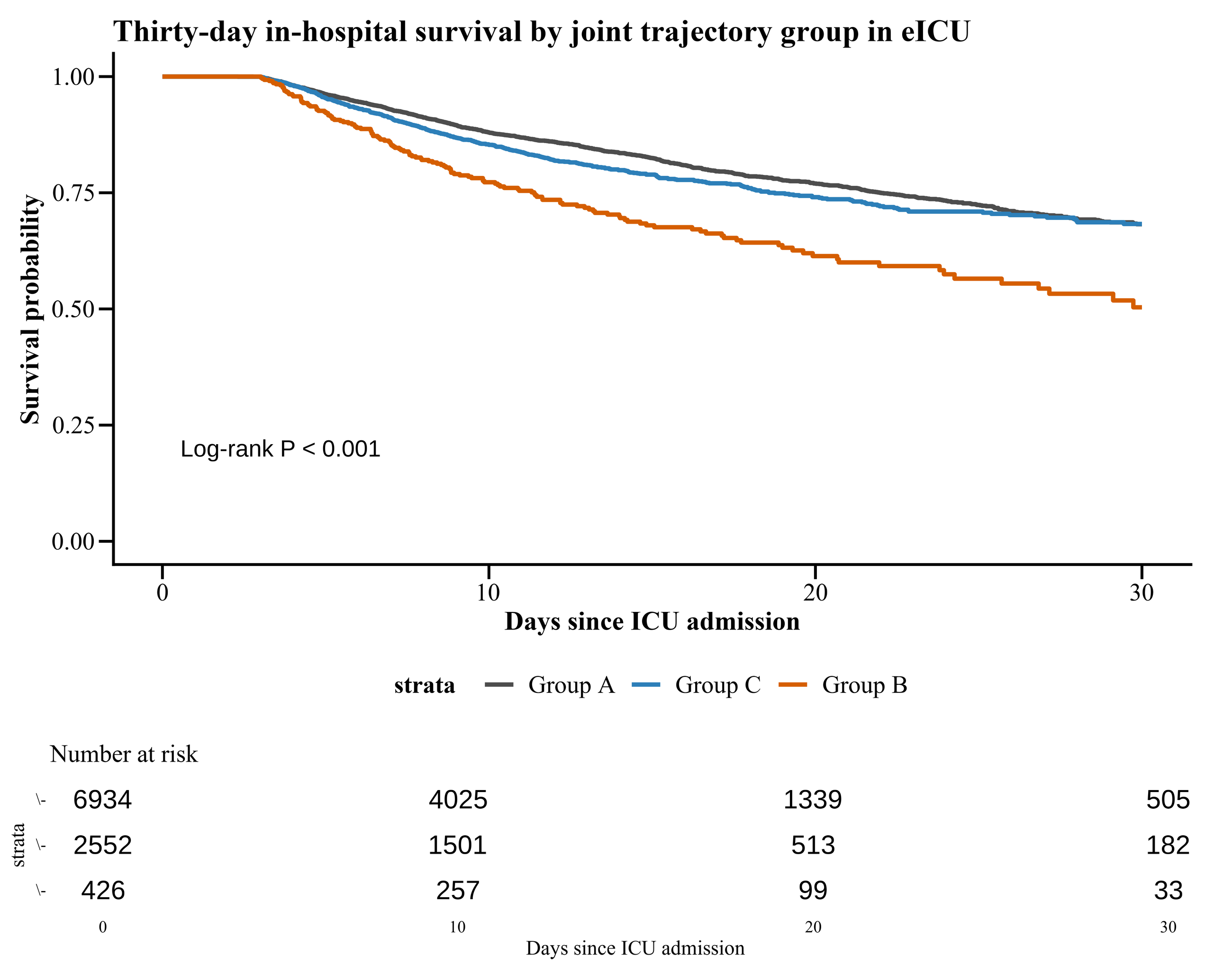


**Supplementary Figure S3. Restricted cubic spline analyses for hospital mortality in MIMIC-IV and eICU.**


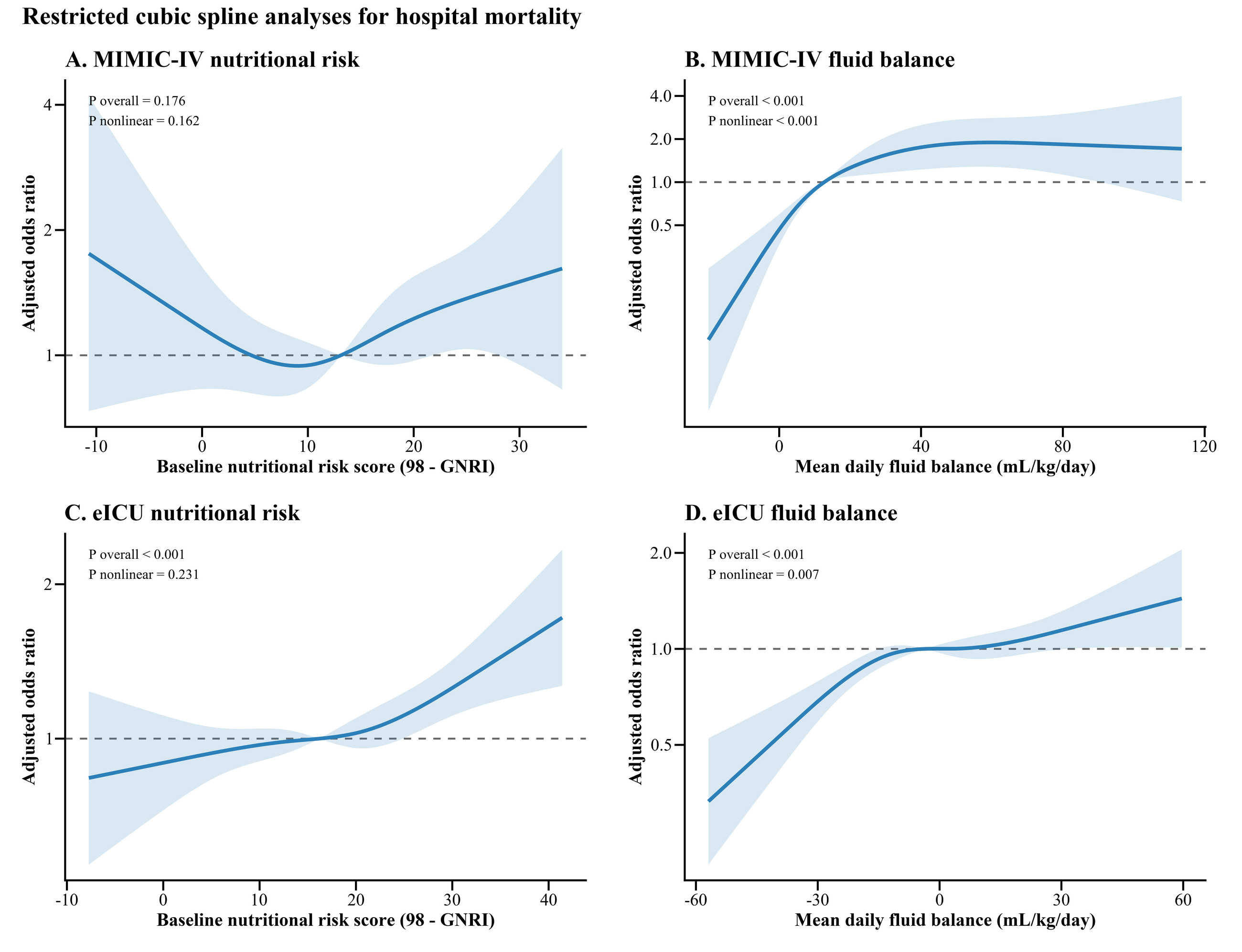


**Supplementary Figure S4. Four-class trajectory sensitivity analyses in MIMIC-IV and eICU.**


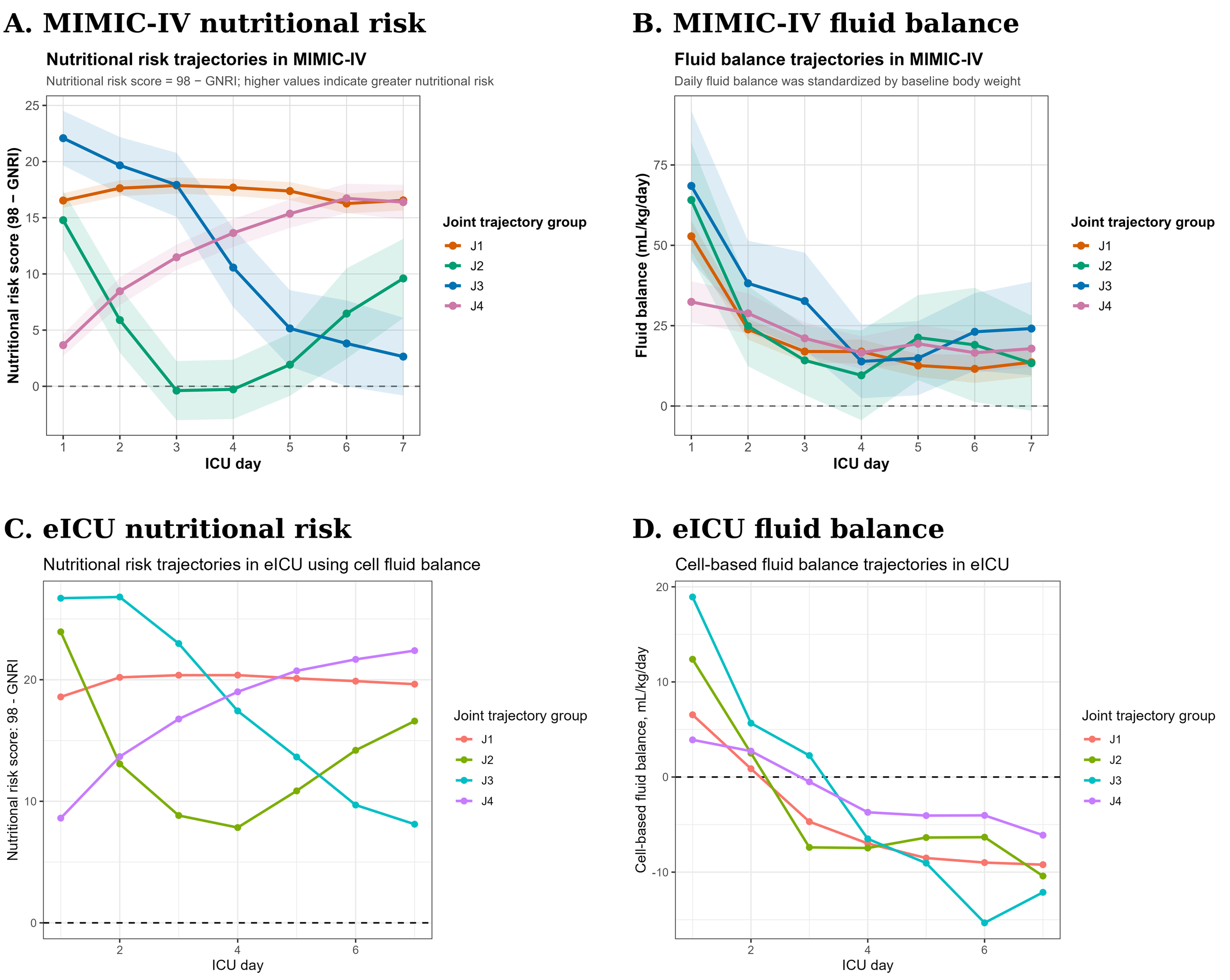


**Supplementary Figure S5A. Subgroup analyses for Group C versus Group A in MIMIC-IV.**


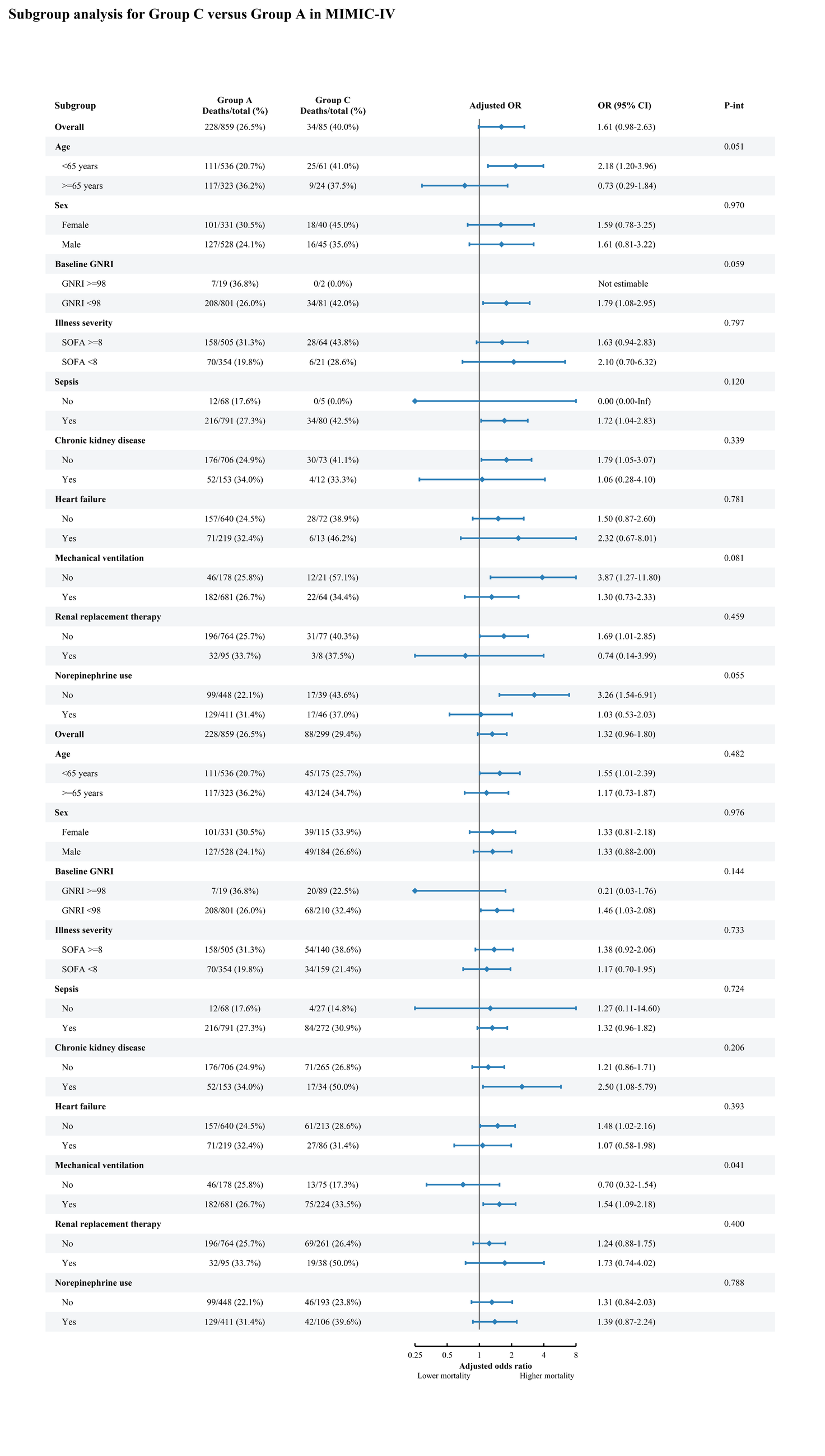


**Supplementary Figure S5B. Subgroup analyses for Group C versus Group A in eICU.**


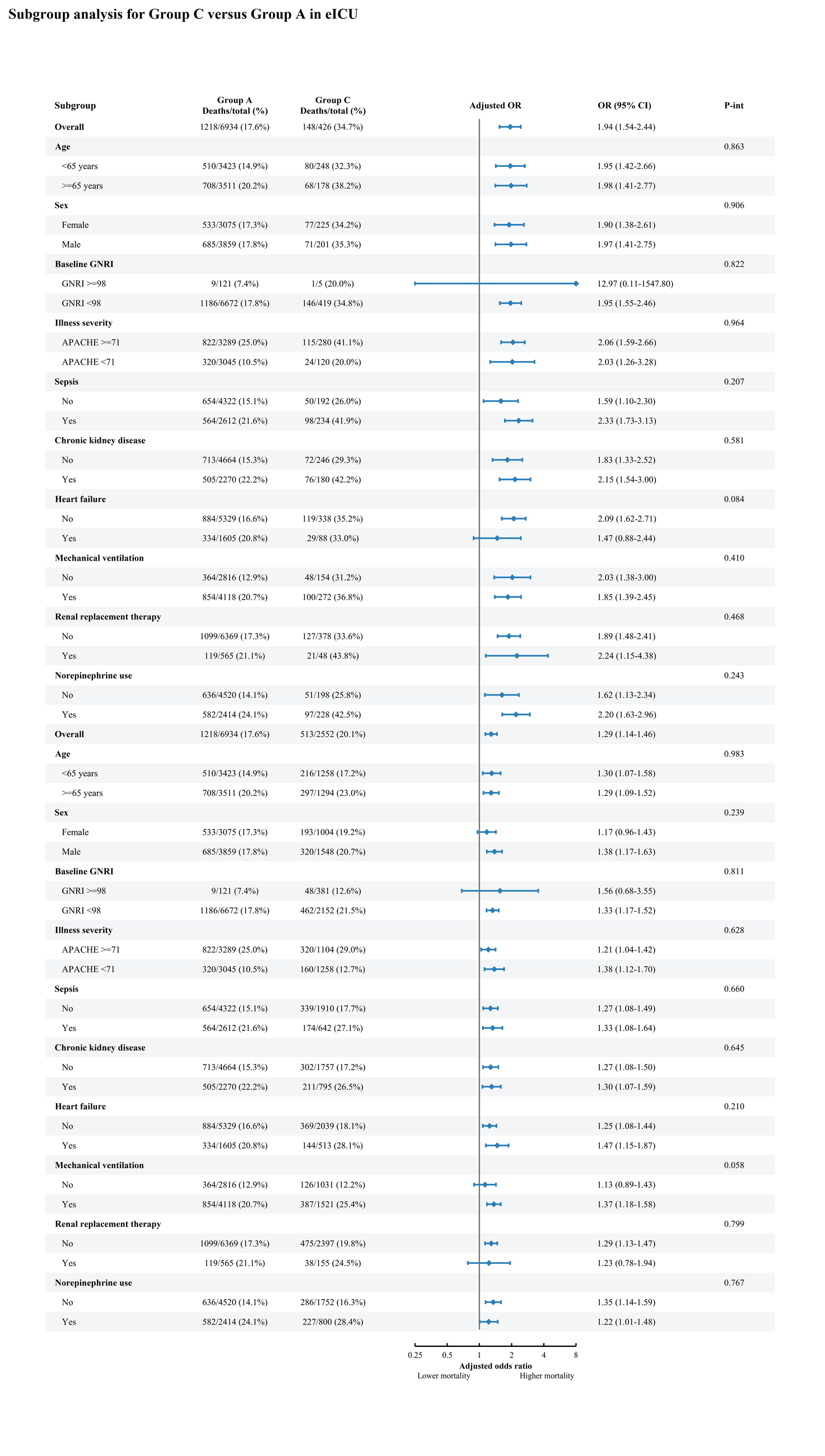


**Supplementary Figure S6. Day-7 landmark analysis.**


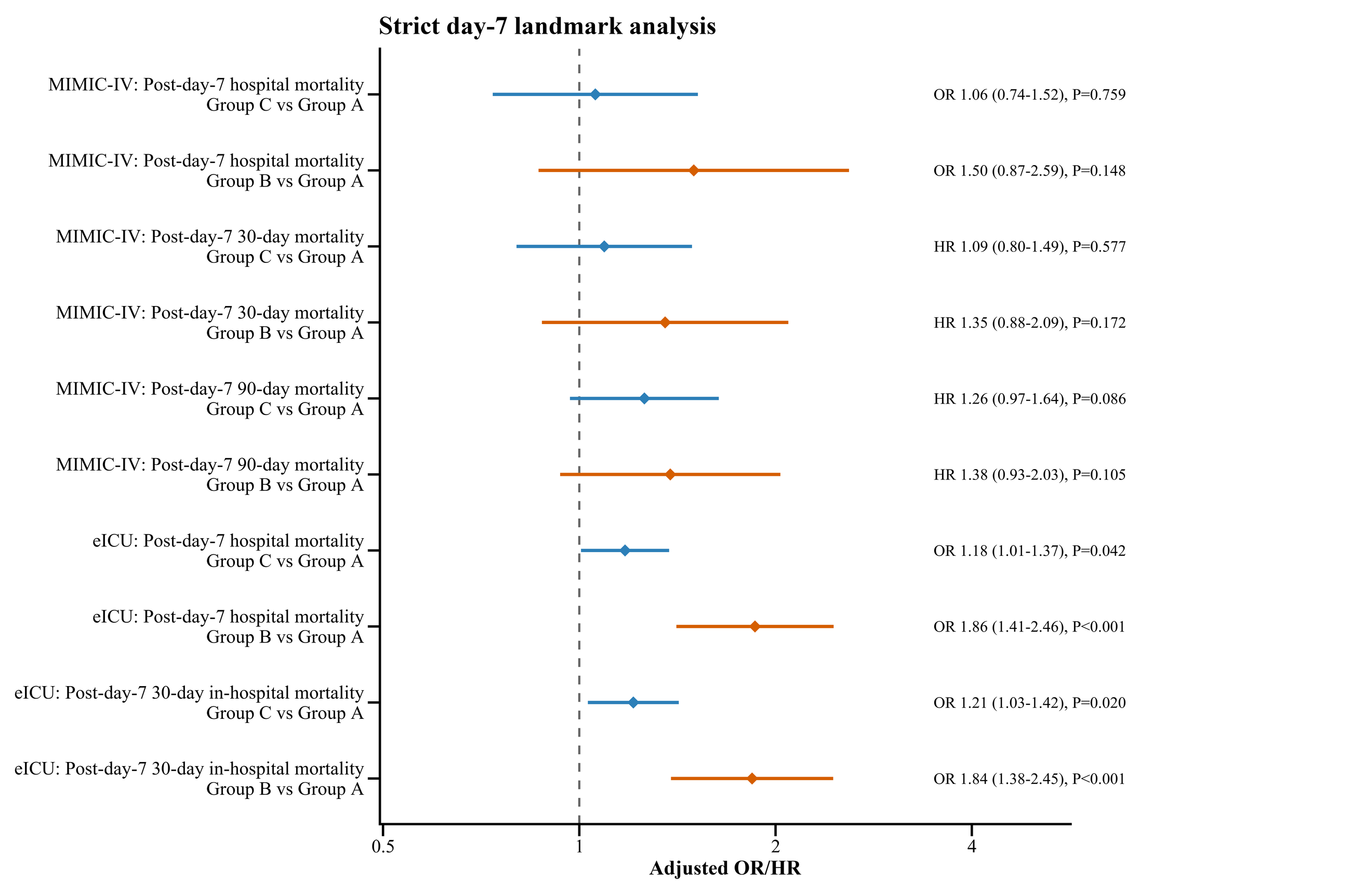


**Supplementary Figure S7. Day-3 short-window joint trajectories.**


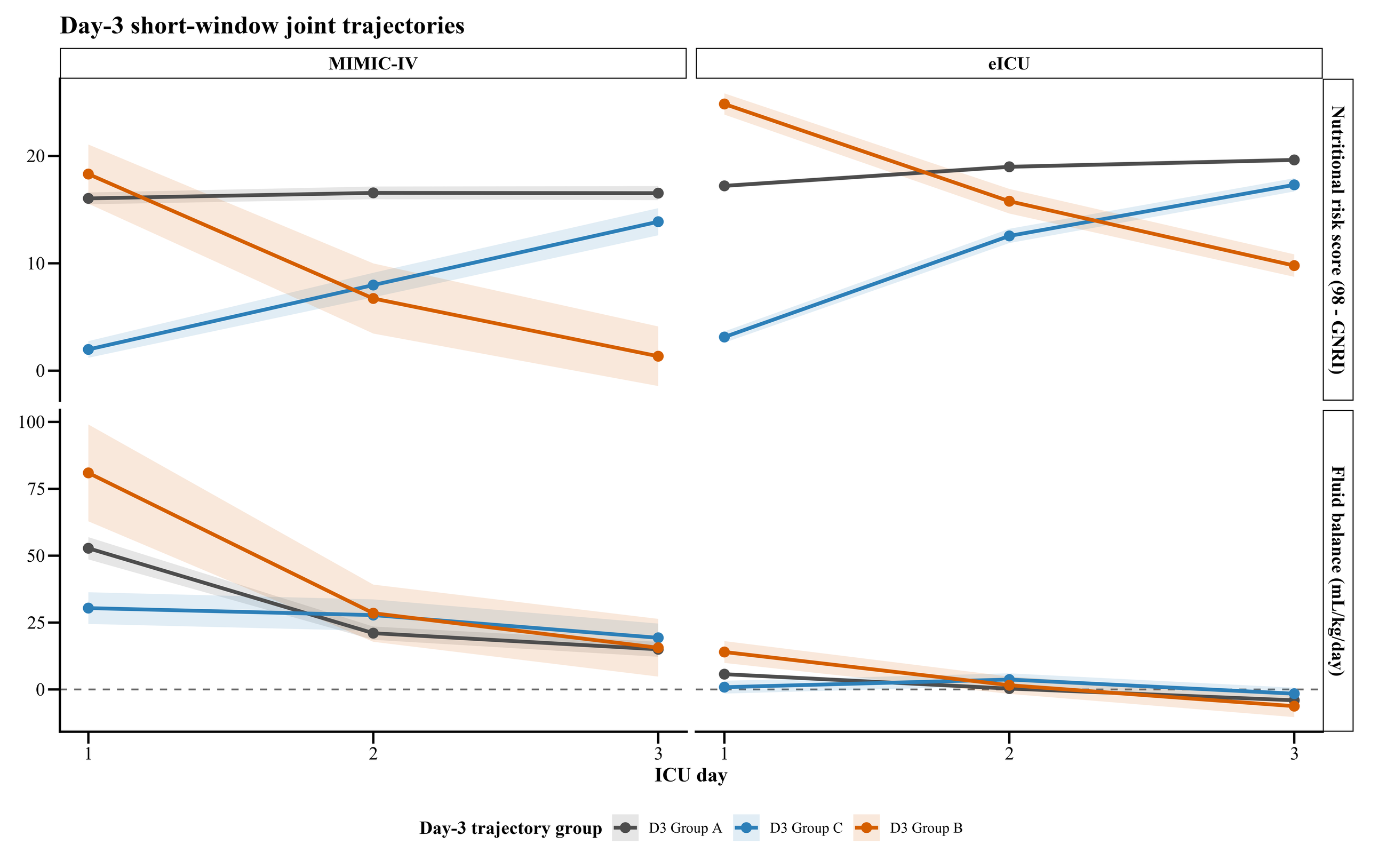


**Supplementary Figure S8. Day-3 short-window landmark analysis.**


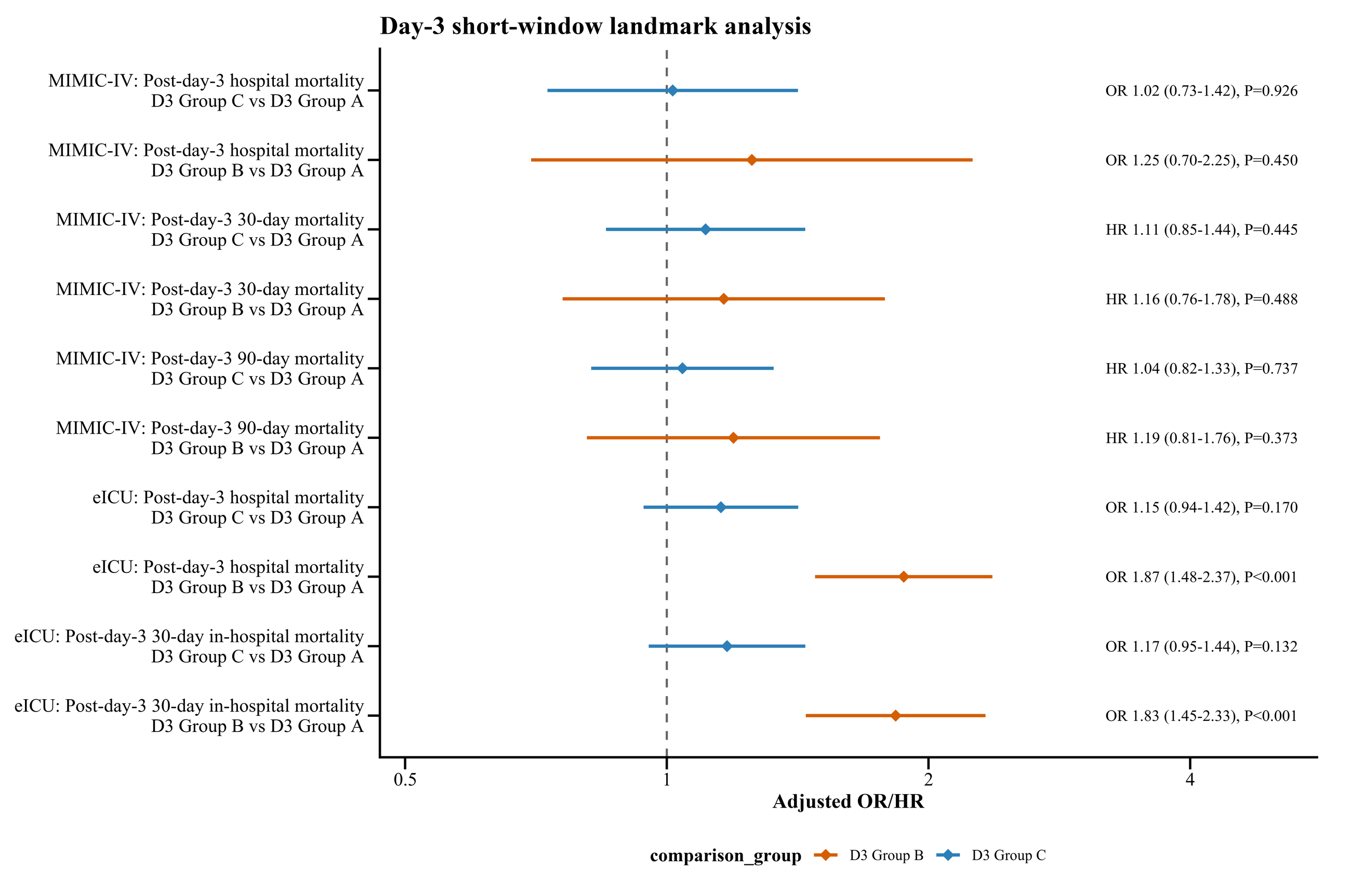


**Supplementary Figure S9. Extended adjustment for baseline GNRI, albumin, and early fluid balance.**


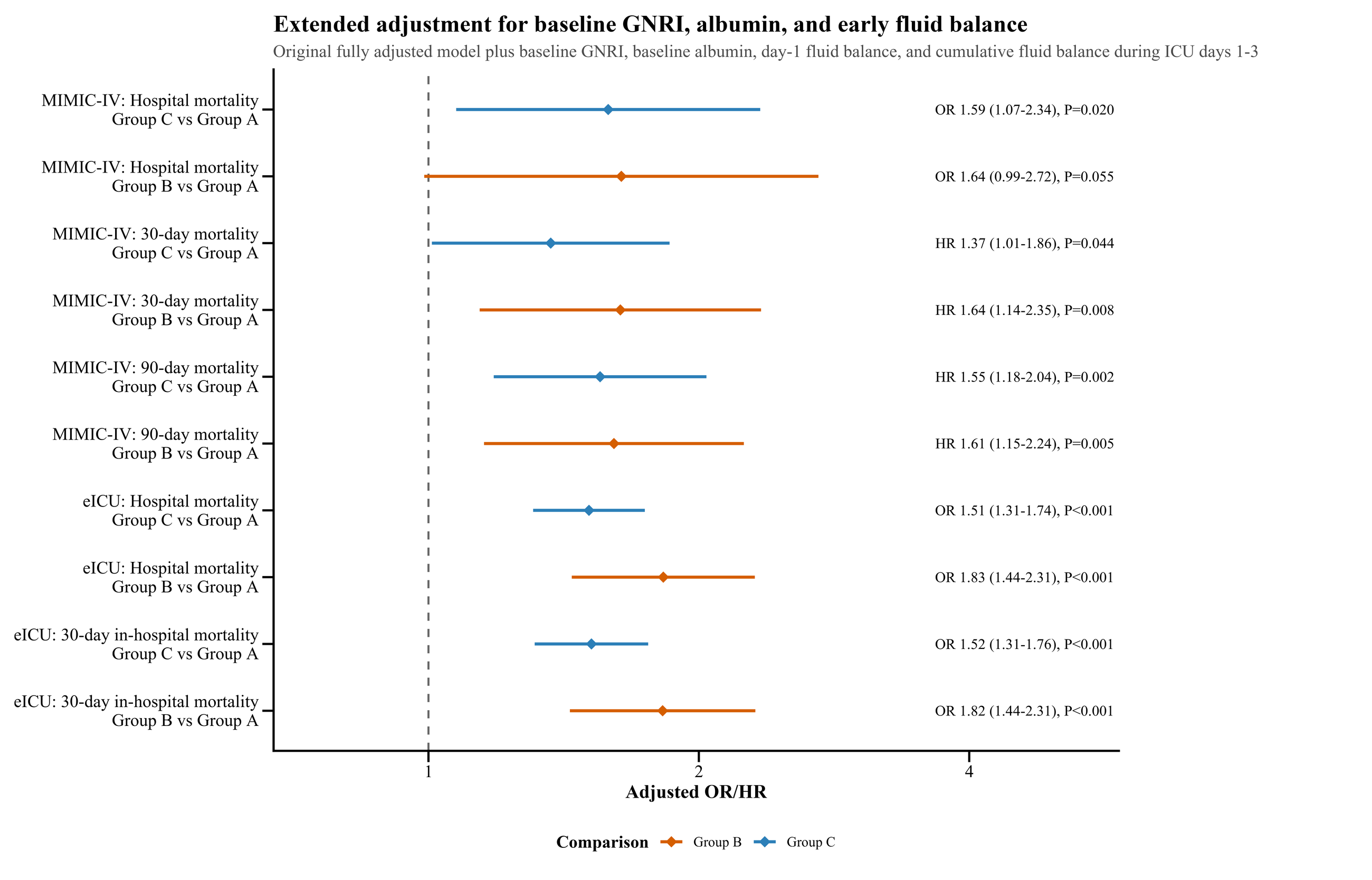


**Supplementary Figure S10. Calibration curves for incremental prediction models.**


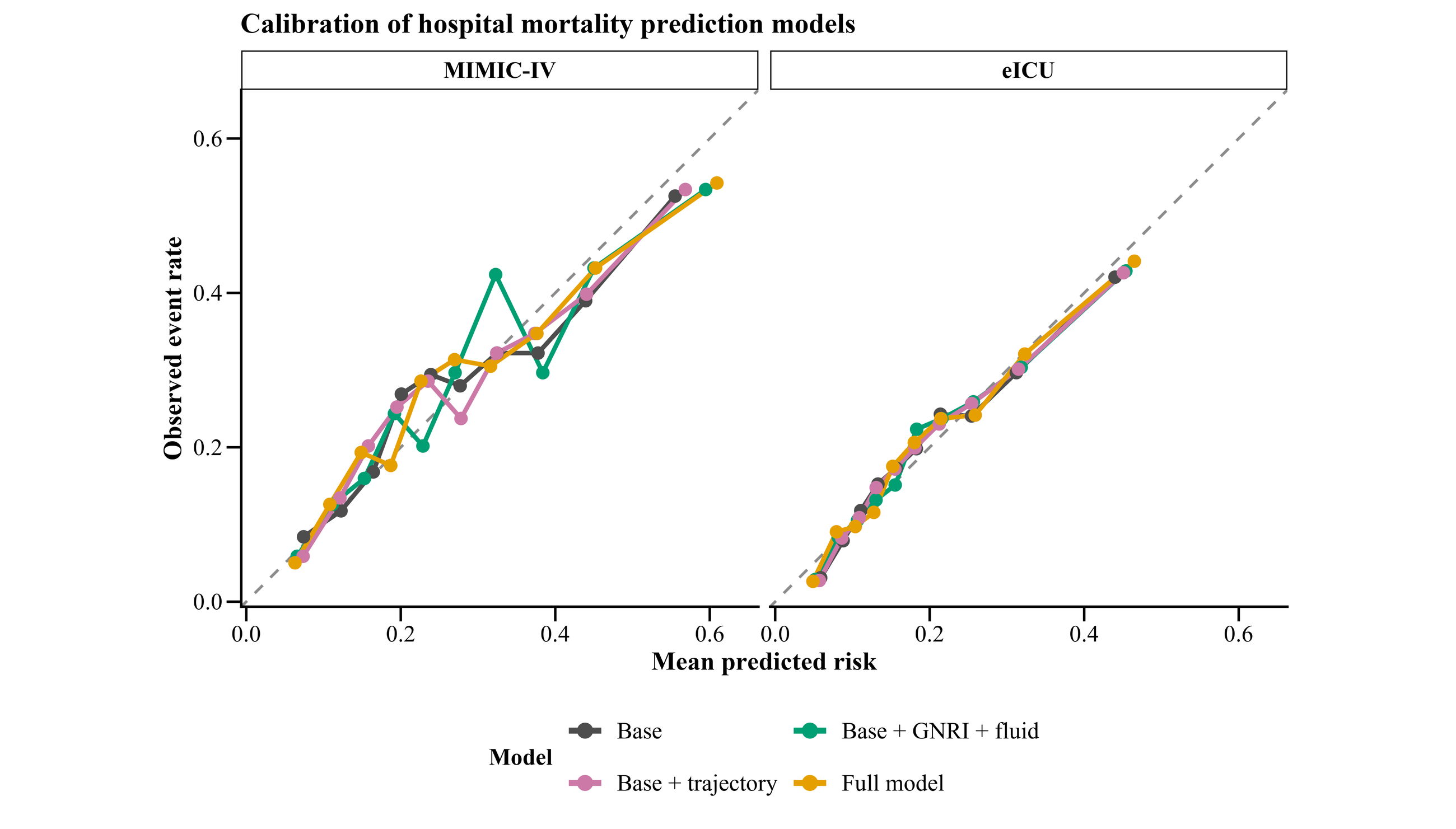


**Supplementary Figure S11. Decision curve analysis for hospital mortality.**


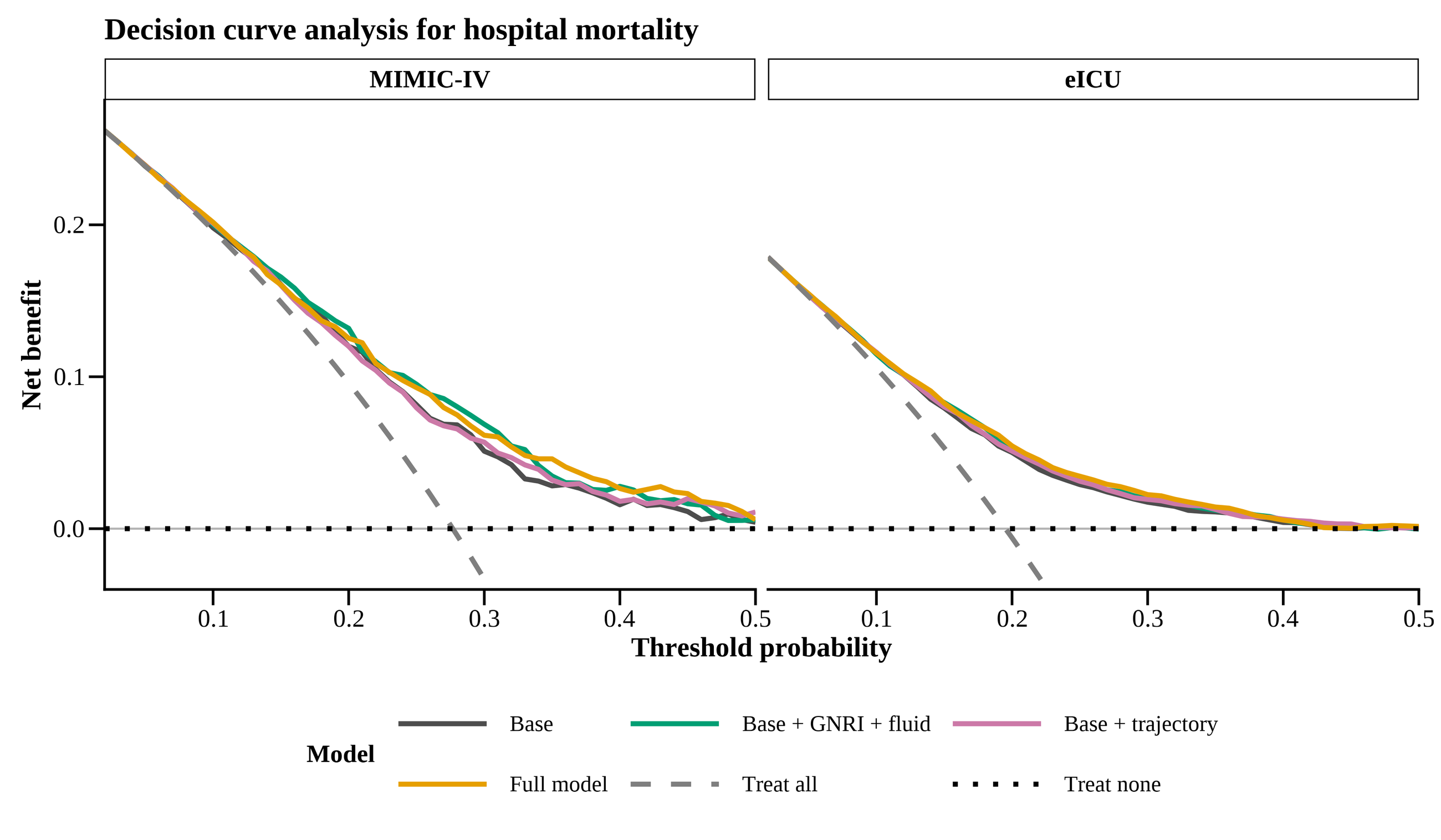


**Supplementary Figure S12. Incremental predictive value of trajectory information.**


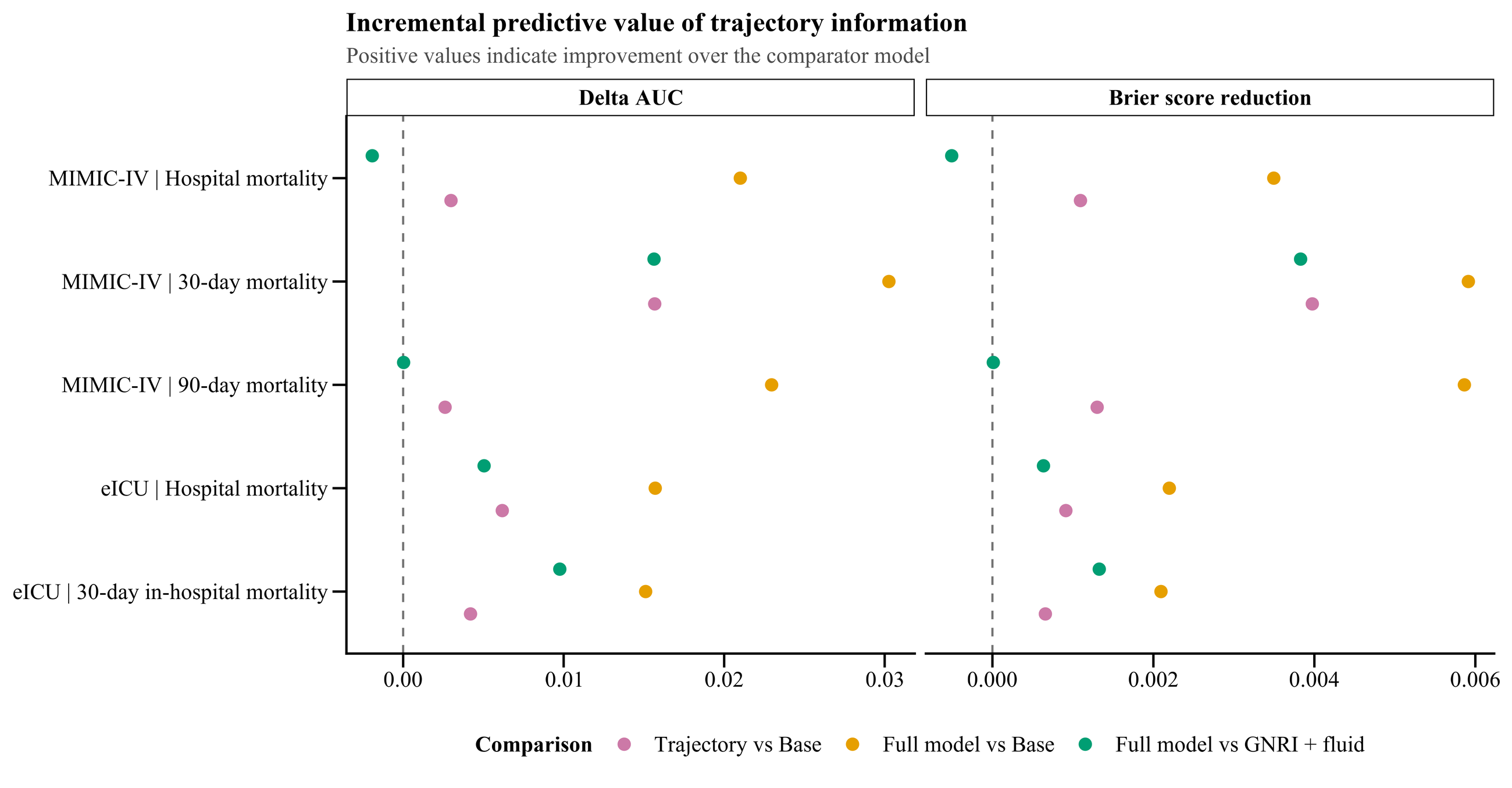


**2 Supplementary Tables**

**Supplementary Table S1. Missingness of candidate baseline covariates.**

| **database_source** | **n_stays** | **variable** | **missing_percent** |
| --- | --- | --- | --- |
| MIMIC-IV | 1243 | apache_score | 100.0 |
| MIMIC-IV | 1243 | acute_physiology_score | 100.0 |
| MIMIC-IV | 1243 | gnri_24h | 22.6 |
| MIMIC-IV | 1243 | nutrition_risk_24h | 22.6 |
| MIMIC-IV | 1243 | albumin_24h_gdl | 22.6 |
| MIMIC-IV | 1243 | lactate_24h | 12.3 |
| MIMIC-IV | 1243 | temperature_c_24h | 4.7 |
| MIMIC-IV | 1243 | gnri_baseline | 3.3 |
| MIMIC-IV | 1243 | nutrition_risk_baseline | 3.3 |
| MIMIC-IV | 1243 | albumin_baseline_gdl | 3.3 |
| MIMIC-IV | 1243 | sbp_24h | 0.9 |
| MIMIC-IV | 1243 | dbp_24h | 0.9 |
| MIMIC-IV | 1243 | wbc_24h | 0.3 |
| MIMIC-IV | 1243 | anion_gap_24h | 0.3 |
| MIMIC-IV | 1243 | hemoglobin_24h | 0.2 |
| MIMIC-IV | 1243 | bun_24h | 0.2 |
| MIMIC-IV | 1243 | platelet_24h | 0.2 |
| MIMIC-IV | 1243 | sodium_24h | 0.2 |
| MIMIC-IV | 1243 | creatinine_24h | 0.2 |
| MIMIC-IV | 1243 | potassium_24h | 0.1 |
| MIMIC-IV | 1243 | age | 0.0 |
| MIMIC-IV | 1243 | sex | 0.0 |
| MIMIC-IV | 1243 | race_ethnicity | 0.0 |
| MIMIC-IV | 1243 | height_cm | 0.0 |
| MIMIC-IV | 1243 | weight_kg | 0.0 |
| MIMIC-IV | 1243 | bmi | 0.0 |
| MIMIC-IV | 1243 | heart_rate_24h | 0.0 |
| MIMIC-IV | 1243 | resp_rate_24h | 0.0 |
| MIMIC-IV | 1243 | mbp_24h | 0.0 |
| MIMIC-IV | 1243 | spo2_24h | 0.0 |
| MIMIC-IV | 1243 | hf | 0.0 |
| MIMIC-IV | 1243 | af | 0.0 |
| MIMIC-IV | 1243 | hypertension | 0.0 |
| MIMIC-IV | 1243 | diabetes | 0.0 |
| MIMIC-IV | 1243 | ckd | 0.0 |
| MIMIC-IV | 1243 | sepsis | 0.0 |
| MIMIC-IV | 1243 | ventilation_24h | 0.0 |
| MIMIC-IV | 1243 | rrt_24h | 0.0 |
| MIMIC-IV | 1243 | crrt_24h | 0.0 |
| MIMIC-IV | 1243 | furosemide_24h | 0.0 |
| MIMIC-IV | 1243 | spironolactone_24h | 0.0 |
| MIMIC-IV | 1243 | norepinephrine_24h | 0.0 |
| MIMIC-IV | 1243 | sofa | 0.0 |
| MIMIC-IV | 1243 | charlson | 0.0 |
| MIMIC-IV | 1243 | sapsii | 0.0 |
| MIMIC-IV | 1243 | oasis | 0.0 |
| eICU | 9912 | temperature_c_24h | 86.0 |
| eICU | 9912 | lactate_24h | 49.8 |
| eICU | 9912 | predicted_hospital_mortality | 37.8 |
| eICU | 9912 | anion_gap_24h | 16.7 |
| eICU | 9912 | gnri_24h | 15.2 |
| eICU | 9912 | nutrition_risk_24h | 15.2 |
| eICU | 9912 | albumin_24h_gdl | 15.2 |
| eICU | 9912 | apache_score | 8.2 |
| eICU | 9912 | acute_physiology_score | 8.2 |
| eICU | 9912 | resp_rate_24h | 7.0 |
| eICU | 9912 | platelet_24h | 4.6 |
| eICU | 9912 | wbc_24h | 4.3 |
| eICU | 9912 | hemoglobin_24h | 3.8 |
| eICU | 9912 | creatinine_24h | 2.3 |
| eICU | 9912 | bun_24h | 2.2 |
| eICU | 9912 | sodium_24h | 2.0 |
| eICU | 9912 | potassium_24h | 1.8 |
| eICU | 9912 | spo2_24h | 1.8 |
| eICU | 9912 | gnri_baseline | 1.6 |
| eICU | 9912 | nutrition_risk_baseline | 1.6 |
| eICU | 9912 | albumin_baseline_gdl | 1.6 |
| eICU | 9912 | heart_rate_24h | 0.9 |
| eICU | 9912 | sbp_24h | 0.9 |
| eICU | 9912 | dbp_24h | 0.9 |
| eICU | 9912 | mbp_24h | 0.9 |
| eICU | 9912 | bmi | 0.1 |
| eICU | 9912 | age | 0.0 |
| eICU | 9912 | sex | 0.0 |
| eICU | 9912 | race_ethnicity | 0.0 |
| eICU | 9912 | height_cm | 0.0 |
| eICU | 9912 | weight_kg | 0.0 |
| eICU | 9912 | hf | 0.0 |
| eICU | 9912 | af | 0.0 |
| eICU | 9912 | hypertension | 0.0 |
| eICU | 9912 | diabetes | 0.0 |
| eICU | 9912 | ckd | 0.0 |
| eICU | 9912 | sepsis | 0.0 |
| eICU | 9912 | ventilation_24h | 0.0 |
| eICU | 9912 | rrt_24h | 0.0 |
| eICU | 9912 | crrt_24h | 0.0 |
| eICU | 9912 | furosemide_24h | 0.0 |
| eICU | 9912 | spironolactone_24h | 0.0 |
| eICU | 9912 | norepinephrine_24h | 0.0 |

Note. Values indicate the percentage of missing observations among the final main analysis cohort within each database.

**Supplementary Table S2. Characteristics of included and excluded patients.**

Values are median [interquartile range] (available n) or event/available n (%). P values compare excluded and included patients within each database. This table evaluates potential selection bias introduced by the requirement for sufficient repeated GNRI-derived nutritional-risk and fluid-balance measurements.

**Panel A. MIMIC-IV**

| **Variable** | **Excluded (n=42676)** | **Included (n=1243)** | **P value** |
| --- | --- | --- | --- |
| **Demographics** | | | |
| Age, years | 65.0 [53.0; 76.0] (n=42676) | 60.0 [48.0; 71.0] (n=1243) | <0.001 |
| Male sex | 24553/42676 (57.5%) | 757/1243 (60.9%) | 0.018 |
| Body mass index, kg/m² | 27.7 [24.2; 32.1] (n=21589) | 28.4 [24.5; 33.4] (n=1243) | <0.001 |
| Body weight, kg | 79.1 [66.6; 94.0] (n=42426) | 82.9 [69.8; 98.6] (n=1243) | <0.001 |
| **Nutritional-fluid baseline variables** | | | |
| Baseline GNRI | 87.9 [81.1; 95.2] (n=7099) | 84.9 [77.4; 90.8] (n=1202) | <0.001 |
| Baseline nutritional risk score | 10.1 [2.8; 16.9] (n=7099) | 13.1 [7.2; 20.6] (n=1202) | <0.001 |
| Baseline albumin, g/dL | 3.20 [2.70; 3.70] (n=14358) | 2.90 [2.50; 3.30] (n=1202) | <0.001 |
| Day-1 fluid balance, mL/kg/day | 16.0 [-0.7; 43.1] (n=36950) | 31.8 [7.1; 80.1] (n=1224) | <0.001 |
| Mean fluid balance, days 1-3, mL/kg/day | 9.3 [-1.5; 23.0] (n=37167) | 22.8 [5.4; 49.0] (n=1243) | <0.001 |
| Cumulative fluid balance, days 1-3, mL/kg | 11.5 [0.0; 42.8] (n=42426) | 68.1 [16.5; 148.2] (n=1243) | <0.001 |
| **Clinical course** | | | |
| ICU length of stay, days | 1.9 [1.1; 3.6] (n=42667) | 9.2 [6.3; 15.6] (n=1243) | <0.001 |
| Hospital length of stay, days | 6.5 [3.8; 11.0] (n=42676) | 17.5 [11.0; 27.8] (n=1243) | <0.001 |
| **Physiology and laboratory data** | | | |
| Heart rate, beats/min | 82.1 [72.8; 93.0] (n=42614) | 90.5 [78.3; 104.4] (n=1243) | <0.001 |
| Respiratory rate, breaths/min | 18.2 [16.3; 20.8] (n=42529) | 20.5 [17.9; 24.0] (n=1243) | <0.001 |
| Mean blood pressure, mmHg | 78.5 [72.2; 86.6] (n=42579) | 76.6 [71.0; 83.4] (n=1243) | <0.001 |
| Creatinine, mg/dL | 0.90 [0.70; 1.20] (n=41232) | 1.17 [0.80; 2.08] (n=1241) | <0.001 |
| Blood urea nitrogen, mg/dL | 16.0 [12.0; 24.5] (n=41221) | 22.2 [14.7; 39.7] (n=1240) | <0.001 |
| Lactate, mmol/L | 1.81 [1.33; 2.53] (n=22533) | 2.17 [1.43; 3.61] (n=1090) | <0.001 |
| **Comorbidities** | | | |
| Heart failure | 8323/42676 (19.5%) | 318/1243 (25.6%) | <0.001 |
| Atrial fibrillation | 11060/42676 (25.9%) | 363/1243 (29.2%) | 0.009 |
| Hypertension | 25842/42676 (60.6%) | 667/1243 (53.7%) | <0.001 |
| Diabetes mellitus | 10771/42676 (25.2%) | 311/1243 (25.0%) | 0.861 |
| Chronic kidney disease | 5755/42676 (13.5%) | 199/1243 (16.0%) | 0.010 |
| Sepsis | 17149/42676 (40.2%) | 1135/1243 (91.3%) | <0.001 |
| **Treatments within 24 h** | | | |
| Mechanical ventilation | 15979/42676 (37.4%) | 937/1243 (75.4%) | <0.001 |
| Renal replacement therapy | 1154/42676 (2.7%) | 141/1243 (11.3%) | <0.001 |
| Furosemide use | 9915/42676 (23.2%) | 333/1243 (26.8%) | 0.003 |
| Spironolactone use | 453/42676 (1.1%) | 17/1243 (1.4%) | 0.301 |
| Norepinephrine use | 4917/42676 (11.5%) | 563/1243 (45.3%) | <0.001 |
| **Outcomes** | | | |
| Hospital mortality | 4285/42676 (10.0%) | 350/1243 (28.2%) | <0.001 |
| 30-day mortality / 30-day in-hospital mortality | 5347/42676 (12.5%) | 357/1243 (28.7%) | <0.001 |
| **Severity scores** | | | |
| SOFA score | 3.0 [2.0; 6.0] (n=42676) | 8.0 [5.0; 11.0] (n=1243) | <0.001 |
| SAPS II score | 32.0 [24.0; 41.0] (n=42676) | 43.0 [34.0; 55.0] (n=1243) | <0.001 |
| OASIS score | 30.0 [24.0; 36.0] (n=42676) | 37.0 [32.0; 44.0] (n=1243) | <0.001 |
| Charlson comorbidity index | 4.0 [2.0; 6.0] (n=42676) | 4.0 [2.5; 6.0] (n=1243) | <0.001 |
| **Outcomes** | | | |
| 90-day mortality | 6875/42676 (16.1%) | 439/1243 (35.3%) | <0.001 |

**Panel B. eICU**

| **Variable** | **Excluded (n=128956)** | **Included (n=9912)** | **P value** |
| --- | --- | --- | --- |
| **Demographics** | | | |
| Age, years | 65.0 [53.0; 77.0] (n=128956) | 65.0 [53.0; 75.0] (n=9912) | 0.001 |
| Male sex | 69352/128879 (53.8%) | 5608/9912 (56.6%) | <0.001 |
| Body mass index, kg/m² | 27.5 [23.6; 32.7] (n=122495) | 27.8 [23.6; 33.4] (n=9904) | <0.001 |
| Body weight, kg | 80.0 [66.4; 96.6] (n=124196) | 81.3 [67.3; 98.6] (n=9912) | <0.001 |
| **Nutritional-fluid baseline variables** | | | |
| Baseline GNRI | 84.9 [77.4; 92.3] (n=58571) | 81.8 [74.5; 87.9] (n=9750) | <0.001 |
| Baseline nutritional risk score | 13.1 [5.7; 20.6] (n=58571) | 16.2 [10.1; 23.5] (n=9750) | <0.001 |
| Baseline albumin, g/dL | 3.00 [2.50; 3.40] (n=60990) | 2.70 [2.20; 3.20] (n=9750) | <0.001 |
| Day-1 fluid balance, mL/kg/day | -5.0 [-17.3; 9.9] (n=101223) | -0.9 [-15.2; 23.6] (n=9665) | <0.001 |
| Mean fluid balance, days 1-3, mL/kg/day | -5.3 [-16.2; 6.9] (n=104808) | -1.6 [-16.7; 17.9] (n=9887) | <0.001 |
| Cumulative fluid balance, days 1-3, mL/kg | -2.2 [-28.2; 7.0] (n=124196) | -4.3 [-49.3; 52.2] (n=9912) | <0.001 |
| **Clinical course** | | | |
| ICU length of stay, days | 1.5 [0.9; 2.6] (n=128956) | 6.0 [4.2; 9.6] (n=9912) | <0.001 |
| Hospital length of stay, days | 4.7 [2.5; 8.2] (n=128956) | 11.8 [7.8; 18.0] (n=9912) | <0.001 |
| **Physiology and laboratory data** | | | |
| Heart rate, beats/min | 82.2 [71.7; 94.1] (n=124792) | 89.5 [77.8; 102.5] (n=9824) | <0.001 |
| Respiratory rate, breaths/min | 18.6 [16.2; 21.8] (n=115704) | 19.8 [16.8; 23.7] (n=9223) | <0.001 |
| Mean blood pressure, mmHg | 80.6 [72.4; 90.5] (n=124648) | 77.2 [70.3; 86.4] (n=9827) | <0.001 |
| Creatinine, mg/dL | 0.95 [0.73; 1.40] (n=110232) | 1.23 [0.81; 2.12] (n=9683) | <0.001 |
| Blood urea nitrogen, mg/dL | 18.0 [12.0; 29.0] (n=109739) | 24.4 [15.0; 42.0] (n=9690) | <0.001 |
| Lactate, mmol/L | 1.70 [1.10; 2.65] (n=28367) | 2.02 [1.30; 3.30] (n=4979) | <0.001 |
| **Comorbidities** | | | |
| Heart failure | 21348/128956 (16.6%) | 2206/9912 (22.3%) | <0.001 |
| Atrial fibrillation | 18973/128956 (14.7%) | 1829/9912 (18.5%) | <0.001 |
| Hypertension | 67303/128956 (52.2%) | 5195/9912 (52.4%) | 0.672 |
| Diabetes mellitus | 38012/128956 (29.5%) | 3106/9912 (31.3%) | <0.001 |
| Chronic kidney disease | 22076/128956 (17.1%) | 3245/9912 (32.7%) | <0.001 |
| Sepsis | 19132/128956 (14.8%) | 3488/9912 (35.2%) | <0.001 |
| **Treatments within 24 h** | | | |
| Mechanical ventilation | 37957/128956 (29.4%) | 5911/9912 (59.6%) | <0.001 |
| Renal replacement therapy | 5192/128956 (4.0%) | 768/9912 (7.7%) | <0.001 |
| Furosemide use | 16240/128956 (12.6%) | 1827/9912 (18.4%) | <0.001 |
| Spironolactone use | 744/128956 (0.6%) | 80/9912 (0.8%) | 0.004 |
| Norepinephrine use | 13324/128956 (10.3%) | 3442/9912 (34.7%) | <0.001 |
| **Outcomes** | | | |
| Hospital mortality | 11232/128956 (8.7%) | 1879/9912 (19.0%) | <0.001 |
| 30-day mortality / 30-day in-hospital mortality | 11051/128956 (8.6%) | 1788/9912 (18.0%) | <0.001 |
| **Severity scores** | | | |
| APACHE score | 49.0 [35.0; 65.0] (n=106817) | 71.0 [53.0; 92.0] (n=9096) | <0.001 |
| Acute physiology score | 36.0 [26.0; 51.0] (n=106817) | 58.0 [41.0; 79.0] (n=9096) | <0.001 |
| Predicted hospital mortality | 0.139 [0.033; 0.265] (n=45131) | 0.250 [0.146; 0.449] (n=6161) | <0.001 |

**Supplementary Table S3. Trajectory model diagnostics and observation distribution.**

**Panel A. Candidate model-selection diagnostics for day 1-7 joint trajectory models.**

| **Database** | **Classes** | **Polynomial order** | **Link/distribution** | **Random effect** | **Grid starts** | **Convergence** | **AIC** | **BIC** | **Entropy** | **Minimum group %** | **AvePP range** | **OCC range** | **Selected primary** |
| --- | --- | --- | --- | --- | --- | --- | --- | --- | --- | --- | --- | --- | --- |
| MIMIC-IV | 1 | Cubic | Linear link for both outcomes | Random intercept | 0 | 1 | 24103.3 | 24149.4 |  | 100.0 | 1.000-1.000 | NA | No |
| MIMIC-IV | 2 | Cubic | Linear link for both outcomes | Random intercept | 30 | 1 | 23606.8 | 23678.6 | 0.739 | 12.4 | 0.842-0.937 | 2.11-37.61 | No |
| MIMIC-IV | 3 | Cubic | Linear link for both outcomes | Random intercept | 30 | 1 | 23375.1 | 23472.5 | 0.650 | 6.8 | 0.792-0.887 | 2.35-106.53 | Yes |
| MIMIC-IV | 4 | Cubic | Linear link for both outcomes | Random intercept | 30 | 1 | 23181.3 | 23304.3 | 0.705 | 4.7 | 0.783-0.868 | 2.90-131.53 | No |
| MIMIC-IV | 5 | Cubic | Linear link for both outcomes | Random intercept | 30 | 1 | 23104.4 | 23253.0 | 0.700 | 2.3 | 0.758-0.865 | 1.93-277.29 | No |
| eICU | 1 | Cubic | Linear link for both outcomes | Random intercept | 0 | 1 | 212196.6 | 212261.5 |  | 100.0 | 1.000-1.000 | NA | No |
| eICU | 2 | Cubic | Linear link for both outcomes | Random intercept | 30 | 1 | 207121.1 | 207221.9 | 0.784 | 10.6 | 0.852-0.952 | 2.36-48.18 | No |
| eICU | 3 | Cubic | Linear link for both outcomes | Random intercept | 30 | 1 | 204155.1 | 204291.9 | 0.687 | 4.3 | 0.815-0.905 | 2.54-213.23 | Yes |
| eICU | 4 | Cubic | Linear link for both outcomes | Random intercept | 30 | 1 | 202843.8 | 203016.6 | 0.737 | 3.1 | 0.815-0.890 | 2.67-249.17 | No |
| eICU | 5 | Cubic | Linear link for both outcomes | Random intercept | 30 | 1 | 201798.8 | 202007.7 | 0.701 | 1.7 | 0.776-0.890 | 1.70-463.70 | No |

Note. Candidate models with 1-5 classes used cubic time terms, linear links for both outcomes, and a random intercept. Although 4- and 5-class models had lower information criteria, the 3-class model was selected as the primary model based on parsimony, clinical interpretability, reproducibility, and avoidance of unstable over-fragmentation into small classes. MIMIC-IV metrics are extracted from the original primary modeling outputs to maintain consistency with the final 3-class assignments.

**Panel B. Distribution of paired observations used for trajectory modeling.**

| **Cohort** | **n_obs** | **n_stays** | **Proportion (%)** |
| --- | --- | --- | --- |
| MIMIC-IV | 3 | 578 | 46.5 |
| MIMIC-IV | 4 | 350 | 28.2 |
| MIMIC-IV | 5 | 184 | 14.8 |
| MIMIC-IV | 6 | 94 | 7.6 |
| MIMIC-IV | 7 | 37 | 3.0 |
| eICU | 3 | 3061 | 30.9 |
| eICU | 4 | 2267 | 22.9 |
| eICU | 5 | 1654 | 16.7 |
| eICU | 6 | 1297 | 13.1 |
| eICU | 7 | 1633 | 16.5 |

**Panel C. Primary 3-class posterior classification diagnostics.**

| **Database** | **Group** | **n** | **Proportion** | **AvePP** | **Posterior probability, median [IQR]** | **Minimum posterior probability** | **Posterior probability <0.70** | **OCC** | **AvePP flag** | **OCC flag** |
| --- | --- | --- | --- | --- | --- | --- | --- | --- | --- | --- |
| MIMIC-IV | Group A | 859 | 69.1% | 0.84 | 0.884 [0.735-0.963] | 0.399 | 19.6% | 2.35 | Acceptable | Below 5 |
| MIMIC-IV | Group C | 299 | 24.1% | 0.792 | 0.797 [0.625-0.960] | 0.489 | 33.4% | 11.99 | Acceptable | Acceptable |
| MIMIC-IV | Group B | 85 | 6.8% | 0.887 | 0.963 [0.831-0.997] | 0.507 | 15.3% | 106.53 | Acceptable | Acceptable |
| eICU | Group A | 6934 | 70.0% | 0.856 | 0.903 [0.760-0.979] | 0.481 | 17.7% | 2.54 | Acceptable | Below 5 |
| eICU | Group C | 2552 | 25.7% | 0.815 | 0.854 [0.672-0.970] | 0.501 | 28.9% | 12.72 | Acceptable | Acceptable |
| eICU | Group B | 426 | 4.3% | 0.905 | 0.989 [0.859-1.000] | 0.459 | 13.4% | 213.23 | Acceptable | Acceptable |

Note. AvePP values above 0.70 support acceptable average posterior classification. OCC values for the dominant Group A were below 5, reflecting residual classification uncertainty for the largest class; this uncertainty is acknowledged in the limitations and evaluated by a high-confidence posterior probability sensitivity analysis.

**Supplementary Table S4. Four-class sensitivity model classification and clinical outcome summary.**

| **Cohort** | **Class** | **n (%)** | **AvePP** | **OCC** | **Hospital mortality** | **30-day mortality** |
| --- | --- | --- | --- | --- | --- | --- |
| MIMIC-IV | J1 | 809 (65.1%) | 0.844 | 2.90 | 25.6% | 26.5% |
| MIMIC-IV | J2 | 61 (4.9%) | 0.803 | 78.90 | 39.3% | 39.3% |
| MIMIC-IV | J3 | 59 (4.7%) | 0.868 | 131.53 | 44.1% | 44.1% |
| MIMIC-IV | J4 | 314 (25.3%) | 0.783 | 10.67 | 29.6% | 29.6% |
| eICU | J1 | 6822 (68.8%) | 0.855 | 2.67 | 17.3% | 16.3% |
| eICU | J2 | 316 (3.2%) | 0.829 | 147.17 | 33.5% | 31.6% |
| eICU | J3 | 312 (3.1%) | 0.890 | 249.16 | 33.7% | 31.7% |
| eICU | J4 | 2462 (24.8%) | 0.815 | 13.33 | 19.9% | 19.3% |

Note. Four-class models were retained as sensitivity analyses to explore whether smaller high-risk phenotypes were separated from the primary 3-class structure.

**Supplementary Table S5. Detailed subgroup estimates for hospital mortality.**

| **Database** | **Comparison** | **Subgroup** | **Level** | **Group A events/total (%)** | **Comparison events/total (%)** | **Adjusted OR (95% CI)** | **P value** | **P for interaction** |
| --- | --- | --- | --- | --- | --- | --- | --- | --- |
| MIMIC-IV | Group B | Overall | Overall | 228/859 (26.5%) | 34/85 (40.0%) | 1.61 (0.98-2.63) | 0.059 |  |
| **MIMIC-IV \| Group B \| Age (P for interaction = 0.051)** | | | | | | | | |
| MIMIC-IV | Group B | Age | <65 years | 111/536 (20.7%) | 25/61 (41.0%) | 2.18 (1.20-3.96) | 0.010 |  |
| MIMIC-IV | Group B | Age | >=65 years | 117/323 (36.2%) | 9/24 (37.5%) | 0.73 (0.29-1.84) | 0.504 |  |
| **MIMIC-IV \| Group B \| Sex (P for interaction = 0.97)** | | | | | | | | |
| MIMIC-IV | Group B | Sex | Female | 101/331 (30.5%) | 18/40 (45.0%) | 1.59 (0.78-3.25) | 0.205 |  |
| MIMIC-IV | Group B | Sex | Male | 127/528 (24.1%) | 16/45 (35.6%) | 1.61 (0.81-3.22) | 0.177 |  |
| **MIMIC-IV \| Group B \| Baseline GNRI (P for interaction = 0.059)** | | | | | | | | |
| MIMIC-IV | Group B | Baseline GNRI | GNRI >=98 | 7/19 (36.8%) | 0/2 (0.0%) | Not estimable |  |  |
| MIMIC-IV | Group B | Baseline GNRI | GNRI <98 | 208/801 (26.0%) | 34/81 (42.0%) | 1.79 (1.08-2.95) | 0.023 |  |
| **MIMIC-IV \| Group B \| Illness severity (P for interaction = 0.797)** | | | | | | | | |
| MIMIC-IV | Group B | Illness severity | SOFA >=8 | 158/505 (31.3%) | 28/64 (43.8%) | 1.63 (0.94-2.83) | 0.081 |  |
| MIMIC-IV | Group B | Illness severity | SOFA <8 | 70/354 (19.8%) | 6/21 (28.6%) | 2.10 (0.70-6.32) | 0.189 |  |
| **MIMIC-IV \| Group B \| Sepsis (P for interaction = 0.12)** | | | | | | | | |
| MIMIC-IV | Group B | Sepsis | No | 12/68 (17.6%) | 0/5 (0.0%) | 0.00 (0.00-Inf) | 0.995 |  |
| MIMIC-IV | Group B | Sepsis | Yes | 216/791 (27.3%) | 34/80 (42.5%) | 1.72 (1.04-2.83) | 0.035 |  |
| **MIMIC-IV \| Group B \| Chronic kidney disease (P for interaction = 0.339)** | | | | | | | | |
| MIMIC-IV | Group B | Chronic kidney disease | No | 176/706 (24.9%) | 30/73 (41.1%) | 1.79 (1.05-3.07) | 0.034 |  |
| MIMIC-IV | Group B | Chronic kidney disease | Yes | 52/153 (34.0%) | 4/12 (33.3%) | 1.06 (0.28-4.10) | 0.930 |  |
| **MIMIC-IV \| Group B \| Heart failure (P for interaction = 0.781)** | | | | | | | | |
| MIMIC-IV | Group B | Heart failure | No | 157/640 (24.5%) | 28/72 (38.9%) | 1.50 (0.87-2.60) | 0.148 |  |
| MIMIC-IV | Group B | Heart failure | Yes | 71/219 (32.4%) | 6/13 (46.2%) | 2.32 (0.67-8.01) | 0.185 |  |
| **MIMIC-IV \| Group B \| Mechanical ventilation (P for interaction = 0.081)** | | | | | | | | |
| MIMIC-IV | Group B | Mechanical ventilation | No | 46/178 (25.8%) | 12/21 (57.1%) | 3.87 (1.27-11.80) | 0.017 |  |
| MIMIC-IV | Group B | Mechanical ventilation | Yes | 182/681 (26.7%) | 22/64 (34.4%) | 1.30 (0.73-2.33) | 0.369 |  |
| **MIMIC-IV \| Group B \| Renal replacement therapy (P for interaction = 0.459)** | | | | | | | | |
| MIMIC-IV | Group B | Renal replacement therapy | No | 196/764 (25.7%) | 31/77 (40.3%) | 1.69 (1.01-2.85) | 0.047 |  |
| MIMIC-IV | Group B | Renal replacement therapy | Yes | 32/95 (33.7%) | 3/8 (37.5%) | 0.74 (0.14-3.99) | 0.726 |  |
| **MIMIC-IV \| Group B \| Norepinephrine use (P for interaction = 0.055)** | | | | | | | | |
| MIMIC-IV | Group B | Norepinephrine use | No | 99/448 (22.1%) | 17/39 (43.6%) | 3.26 (1.54-6.91) | 0.002 |  |
| MIMIC-IV | Group B | Norepinephrine use | Yes | 129/411 (31.4%) | 17/46 (37.0%) | 1.03 (0.53-2.03) | 0.927 |  |
| MIMIC-IV | Group C | Overall | Overall | 228/859 (26.5%) | 88/299 (29.4%) | 1.32 (0.96-1.80) | 0.084 |  |
| **MIMIC-IV \| Group C \| Age (P for interaction = 0.482)** | | | | | | | | |
| MIMIC-IV | Group C | Age | <65 years | 111/536 (20.7%) | 45/175 (25.7%) | 1.55 (1.01-2.39) | 0.047 |  |
| MIMIC-IV | Group C | Age | >=65 years | 117/323 (36.2%) | 43/124 (34.7%) | 1.17 (0.73-1.87) | 0.520 |  |
| **MIMIC-IV \| Group C \| Sex (P for interaction = 0.976)** | | | | | | | | |
| MIMIC-IV | Group C | Sex | Female | 101/331 (30.5%) | 39/115 (33.9%) | 1.33 (0.81-2.18) | 0.260 |  |
| MIMIC-IV | Group C | Sex | Male | 127/528 (24.1%) | 49/184 (26.6%) | 1.33 (0.88-2.00) | 0.173 |  |
| **MIMIC-IV \| Group C \| Baseline GNRI (P for interaction = 0.144)** | | | | | | | | |
| MIMIC-IV | Group C | Baseline GNRI | GNRI >=98 | 7/19 (36.8%) | 20/89 (22.5%) | 0.21 (0.03-1.76) | 0.151 |  |
| MIMIC-IV | Group C | Baseline GNRI | GNRI <98 | 208/801 (26.0%) | 68/210 (32.4%) | 1.46 (1.03-2.08) | 0.033 |  |
| **MIMIC-IV \| Group C \| Illness severity (P for interaction = 0.733)** | | | | | | | | |
| MIMIC-IV | Group C | Illness severity | SOFA >=8 | 158/505 (31.3%) | 54/140 (38.6%) | 1.38 (0.92-2.06) | 0.123 |  |
| MIMIC-IV | Group C | Illness severity | SOFA <8 | 70/354 (19.8%) | 34/159 (21.4%) | 1.17 (0.70-1.95) | 0.538 |  |
| **MIMIC-IV \| Group C \| Sepsis (P for interaction = 0.724)** | | | | | | | | |
| MIMIC-IV | Group C | Sepsis | No | 12/68 (17.6%) | 4/27 (14.8%) | 1.27 (0.11-14.60) | 0.850 |  |
| MIMIC-IV | Group C | Sepsis | Yes | 216/791 (27.3%) | 84/272 (30.9%) | 1.32 (0.96-1.82) | 0.091 |  |
| **MIMIC-IV \| Group C \| Chronic kidney disease (P for interaction = 0.206)** | | | | | | | | |
| MIMIC-IV | Group C | Chronic kidney disease | No | 176/706 (24.9%) | 71/265 (26.8%) | 1.21 (0.86-1.71) | 0.278 |  |
| MIMIC-IV | Group C | Chronic kidney disease | Yes | 52/153 (34.0%) | 17/34 (50.0%) | 2.50 (1.08-5.79) | 0.032 |  |
| **MIMIC-IV \| Group C \| Heart failure (P for interaction = 0.393)** | | | | | | | | |
| MIMIC-IV | Group C | Heart failure | No | 157/640 (24.5%) | 61/213 (28.6%) | 1.48 (1.02-2.16) | 0.039 |  |
| MIMIC-IV | Group C | Heart failure | Yes | 71/219 (32.4%) | 27/86 (31.4%) | 1.07 (0.58-1.98) | 0.820 |  |
| **MIMIC-IV \| Group C \| Mechanical ventilation (P for interaction = 0.041)** | | | | | | | | |
| MIMIC-IV | Group C | Mechanical ventilation | No | 46/178 (25.8%) | 13/75 (17.3%) | 0.70 (0.32-1.54) | 0.380 |  |
| MIMIC-IV | Group C | Mechanical ventilation | Yes | 182/681 (26.7%) | 75/224 (33.5%) | 1.54 (1.09-2.18) | 0.015 |  |
| **MIMIC-IV \| Group C \| Renal replacement therapy (P for interaction = 0.4)** | | | | | | | | |
| MIMIC-IV | Group C | Renal replacement therapy | No | 196/764 (25.7%) | 69/261 (26.4%) | 1.24 (0.88-1.75) | 0.227 |  |
| MIMIC-IV | Group C | Renal replacement therapy | Yes | 32/95 (33.7%) | 19/38 (50.0%) | 1.73 (0.74-4.02) | 0.205 |  |
| **MIMIC-IV \| Group C \| Norepinephrine use (P for interaction = 0.788)** | | | | | | | | |
| MIMIC-IV | Group C | Norepinephrine use | No | 99/448 (22.1%) | 46/193 (23.8%) | 1.31 (0.84-2.03) | 0.229 |  |
| MIMIC-IV | Group C | Norepinephrine use | Yes | 129/411 (31.4%) | 42/106 (39.6%) | 1.39 (0.87-2.24) | 0.168 |  |
| eICU | Group B | Overall | Overall | 1218/6934 (17.6%) | 148/426 (34.7%) | 1.94 (1.54-2.44) | <0.001 |  |
| **eICU \| Group B \| Age (P for interaction = 0.863)** | | | | | | | | |
| eICU | Group B | Age | <65 years | 510/3423 (14.9%) | 80/248 (32.3%) | 1.95 (1.42-2.66) | <0.001 |  |
| eICU | Group B | Age | >=65 years | 708/3511 (20.2%) | 68/178 (38.2%) | 1.98 (1.41-2.77) | <0.001 |  |
| **eICU \| Group B \| Sex (P for interaction = 0.906)** | | | | | | | | |
| eICU | Group B | Sex | Female | 533/3075 (17.3%) | 77/225 (34.2%) | 1.90 (1.38-2.61) | <0.001 |  |
| eICU | Group B | Sex | Male | 685/3859 (17.8%) | 71/201 (35.3%) | 1.97 (1.41-2.75) | <0.001 |  |
| **eICU \| Group B \| Baseline GNRI (P for interaction = 0.822)** | | | | | | | | |
| eICU | Group B | Baseline GNRI | GNRI >=98 | 9/121 (7.4%) | 1/5 (20.0%) | 12.97 (0.11-1547.80) | 0.294 |  |
| eICU | Group B | Baseline GNRI | GNRI <98 | 1186/6672 (17.8%) | 146/419 (34.8%) | 1.95 (1.55-2.46) | <0.001 |  |
| **eICU \| Group B \| Illness severity (P for interaction = 0.964)** | | | | | | | | |
| eICU | Group B | Illness severity | APACHE >=71 | 822/3289 (25.0%) | 115/280 (41.1%) | 2.06 (1.59-2.66) | <0.001 |  |
| eICU | Group B | Illness severity | APACHE <71 | 320/3045 (10.5%) | 24/120 (20.0%) | 2.03 (1.26-3.28) | 0.004 |  |
| **eICU \| Group B \| Sepsis (P for interaction = 0.207)** | | | | | | | | |
| eICU | Group B | Sepsis | No | 654/4322 (15.1%) | 50/192 (26.0%) | 1.59 (1.10-2.30) | 0.015 |  |
| eICU | Group B | Sepsis | Yes | 564/2612 (21.6%) | 98/234 (41.9%) | 2.33 (1.73-3.13) | <0.001 |  |
| **eICU \| Group B \| Chronic kidney disease (P for interaction = 0.581)** | | | | | | | | |
| eICU | Group B | Chronic kidney disease | No | 713/4664 (15.3%) | 72/246 (29.3%) | 1.83 (1.33-2.52) | <0.001 |  |
| eICU | Group B | Chronic kidney disease | Yes | 505/2270 (22.2%) | 76/180 (42.2%) | 2.15 (1.54-3.00) | <0.001 |  |
| **eICU \| Group B \| Heart failure (P for interaction = 0.084)** | | | | | | | | |
| eICU | Group B | Heart failure | No | 884/5329 (16.6%) | 119/338 (35.2%) | 2.09 (1.62-2.71) | <0.001 |  |
| eICU | Group B | Heart failure | Yes | 334/1605 (20.8%) | 29/88 (33.0%) | 1.47 (0.88-2.44) | 0.139 |  |
| **eICU \| Group B \| Mechanical ventilation (P for interaction = 0.41)** | | | | | | | | |
| eICU | Group B | Mechanical ventilation | No | 364/2816 (12.9%) | 48/154 (31.2%) | 2.03 (1.38-3.00) | <0.001 |  |
| eICU | Group B | Mechanical ventilation | Yes | 854/4118 (20.7%) | 100/272 (36.8%) | 1.85 (1.39-2.45) | <0.001 |  |
| **eICU \| Group B \| Renal replacement therapy (P for interaction = 0.468)** | | | | | | | | |
| eICU | Group B | Renal replacement therapy | No | 1099/6369 (17.3%) | 127/378 (33.6%) | 1.89 (1.48-2.41) | <0.001 |  |
| eICU | Group B | Renal replacement therapy | Yes | 119/565 (21.1%) | 21/48 (43.8%) | 2.24 (1.15-4.38) | 0.018 |  |
| **eICU \| Group B \| Norepinephrine use (P for interaction = 0.243)** | | | | | | | | |
| eICU | Group B | Norepinephrine use | No | 636/4520 (14.1%) | 51/198 (25.8%) | 1.62 (1.13-2.34) | 0.010 |  |
| eICU | Group B | Norepinephrine use | Yes | 582/2414 (24.1%) | 97/228 (42.5%) | 2.20 (1.63-2.96) | <0.001 |  |
| eICU | Group C | Overall | Overall | 1218/6934 (17.6%) | 513/2552 (20.1%) | 1.29 (1.14-1.46) | <0.001 |  |
| **eICU \| Group C \| Age (P for interaction = 0.983)** | | | | | | | | |
| eICU | Group C | Age | <65 years | 510/3423 (14.9%) | 216/1258 (17.2%) | 1.30 (1.07-1.58) | 0.007 |  |
| eICU | Group C | Age | >=65 years | 708/3511 (20.2%) | 297/1294 (23.0%) | 1.29 (1.09-1.52) | 0.003 |  |
| **eICU \| Group C \| Sex (P for interaction = 0.239)** | | | | | | | | |
| eICU | Group C | Sex | Female | 533/3075 (17.3%) | 193/1004 (19.2%) | 1.17 (0.96-1.43) | 0.117 |  |
| eICU | Group C | Sex | Male | 685/3859 (17.8%) | 320/1548 (20.7%) | 1.38 (1.17-1.63) | <0.001 |  |
| **eICU \| Group C \| Baseline GNRI (P for interaction = 0.811)** | | | | | | | | |
| eICU | Group C | Baseline GNRI | GNRI >=98 | 9/121 (7.4%) | 48/381 (12.6%) | 1.56 (0.68-3.55) | 0.291 |  |
| eICU | Group C | Baseline GNRI | GNRI <98 | 1186/6672 (17.8%) | 462/2152 (21.5%) | 1.33 (1.17-1.52) | <0.001 |  |
| **eICU \| Group C \| Illness severity (P for interaction = 0.628)** | | | | | | | | |
| eICU | Group C | Illness severity | APACHE >=71 | 822/3289 (25.0%) | 320/1104 (29.0%) | 1.21 (1.04-1.42) | 0.014 |  |
| eICU | Group C | Illness severity | APACHE <71 | 320/3045 (10.5%) | 160/1258 (12.7%) | 1.38 (1.12-1.70) | 0.003 |  |
| **eICU \| Group C \| Sepsis (P for interaction = 0.66)** | | | | | | | | |
| eICU | Group C | Sepsis | No | 654/4322 (15.1%) | 339/1910 (17.7%) | 1.27 (1.08-1.49) | 0.003 |  |
| eICU | Group C | Sepsis | Yes | 564/2612 (21.6%) | 174/642 (27.1%) | 1.33 (1.08-1.64) | 0.008 |  |
| **eICU \| Group C \| Chronic kidney disease (P for interaction = 0.645)** | | | | | | | | |
| eICU | Group C | Chronic kidney disease | No | 713/4664 (15.3%) | 302/1757 (17.2%) | 1.27 (1.08-1.50) | 0.004 |  |
| eICU | Group C | Chronic kidney disease | Yes | 505/2270 (22.2%) | 211/795 (26.5%) | 1.30 (1.07-1.59) | 0.009 |  |
| **eICU \| Group C \| Heart failure (P for interaction = 0.21)** | | | | | | | | |
| eICU | Group C | Heart failure | No | 884/5329 (16.6%) | 369/2039 (18.1%) | 1.25 (1.08-1.44) | 0.003 |  |
| eICU | Group C | Heart failure | Yes | 334/1605 (20.8%) | 144/513 (28.1%) | 1.47 (1.15-1.87) | 0.002 |  |
| **eICU \| Group C \| Mechanical ventilation (P for interaction = 0.058)** | | | | | | | | |
| eICU | Group C | Mechanical ventilation | No | 364/2816 (12.9%) | 126/1031 (12.2%) | 1.13 (0.89-1.43) | 0.315 |  |
| eICU | Group C | Mechanical ventilation | Yes | 854/4118 (20.7%) | 387/1521 (25.4%) | 1.37 (1.18-1.58) | <0.001 |  |
| **eICU \| Group C \| Renal replacement therapy (P for interaction = 0.799)** | | | | | | | | |
| eICU | Group C | Renal replacement therapy | No | 1099/6369 (17.3%) | 475/2397 (19.8%) | 1.29 (1.13-1.47) | <0.001 |  |
| eICU | Group C | Renal replacement therapy | Yes | 119/565 (21.1%) | 38/155 (24.5%) | 1.23 (0.78-1.94) | 0.376 |  |
| **eICU \| Group C \| Norepinephrine use (P for interaction = 0.767)** | | | | | | | | |
| eICU | Group C | Norepinephrine use | No | 636/4520 (14.1%) | 286/1752 (16.3%) | 1.35 (1.14-1.59) | <0.001 |  |
| eICU | Group C | Norepinephrine use | Yes | 582/2414 (24.1%) | 227/800 (28.4%) | 1.22 (1.01-1.48) | 0.044 |  |

Note. Subgroup analyses were exploratory and were not adjusted for multiplicity. Header rows display P values for interaction where estimable. Event columns show events/total (%).

**Supplementary Table S6. Day-7 landmark results.**

| **Database** | **Outcome** | **Comparison** | **Measure** | **Estimate (95% CI)** | **P value** | **n** | **Events** |
| --- | --- | --- | --- | --- | --- | --- | --- |
| MIMIC-IV | Post-day-7 hospital mortality | Group C vs Group A | OR | 1.06 (0.74-1.52) | 0.759 | 1116 | 257 |
| MIMIC-IV | Post-day-7 hospital mortality | Group B vs Group A | OR | 1.50 (0.87-2.59) | 0.148 | 1116 | 257 |
| MIMIC-IV | Post-day-7 30-day mortality | Group C vs Group A | HR | 1.09 (0.80-1.49) | 0.577 | 1116 | 255 |
| MIMIC-IV | Post-day-7 30-day mortality | Group B vs Group A | HR | 1.35 (0.88-2.09) | 0.172 | 1116 | 255 |
| MIMIC-IV | Post-day-7 90-day mortality | Group C vs Group A | HR | 1.26 (0.97-1.64) | 0.086 | 1116 | 335 |
| MIMIC-IV | Post-day-7 90-day mortality | Group B vs Group A | HR | 1.38 (0.93-2.03) | 0.105 | 1116 | 335 |
| eICU | Post-day-7 hospital mortality | Group C vs Group A | OR | 1.18 (1.01-1.37) | 0.042 | 7171 | 1061 |
| eICU | Post-day-7 hospital mortality | Group B vs Group A | OR | 1.86 (1.41-2.46) | <0.001 | 7171 | 1061 |
| eICU | Post-day-7 30-day in-hospital mortality | Group C vs Group A | OR | 1.21 (1.03-1.42) | 0.020 | 7171 | 975 |
| eICU | Post-day-7 30-day in-hospital mortality | Group B vs Group A | OR | 1.84 (1.38-2.45) | <0.001 | 7171 | 975 |

Note. The day-7 landmark analysis included patients alive and still hospitalized at ICU day 7; follow-up started after the landmark.

**Supplementary Table S7. Day-3 short-window trajectory diagnostics.**

**Panel A. Day-3 short-window eligibility and outcome counts.**

| **Database** | **Screened n** | **Day-3 eligible n** | **Post-day-3 hospital deaths** | **Post-day-3 30-day deaths** | **Post-day-3 90-day deaths** |
| --- | --- | --- | --- | --- | --- |
| MIMIC-IV | 43919 | 1393 | 349 | 376 | 463 |
| eICU | 138868 | 11143 | 2013 | 1935 | 0 |

**Panel B. Day-3 short-window trajectory classification diagnostics.**

| **Database** | **Day-3 group** | **Raw class** | **n** | **Proportion** | **Mean posterior probability** | **AIC** | **BIC** | **Convergence** |
| --- | --- | --- | --- | --- | --- | --- | --- | --- |
| MIMIC-IV | D3_Group_A | J1 | 1054 | 75.7% | 0.797 | 15918.5 | 15986.7 | 1 |
| MIMIC-IV | D3_Group_C | J2 | 282 | 20.2% | 0.703 | 15918.5 | 15986.7 | 1 |
| MIMIC-IV | D3_Group_B | J3 | 57 | 4.1% | 0.861 | 15918.5 | 15986.7 | 1 |
| eICU | D3_Group_A | J3 | 9887 | 88.7% | 0.896 | 134667.5 | 134762.6 | 1 |
| eICU | D3_Group_C | J2 | 842 | 7.6% | 0.739 | 134667.5 | 134762.6 | 1 |
| eICU | D3_Group_B | J1 | 414 | 3.7% | 0.857 | 134667.5 | 134762.6 | 1 |

**Supplementary Table S8. Day-3 short-window landmark results.**

| **Database** | **Outcome** | **Comparison** | **Measure** | **Estimate (95% CI)** | **P value** | **n** | **Events** |
| --- | --- | --- | --- | --- | --- | --- | --- |
| MIMIC-IV | Post-day-3 hospital mortality | D3 Group C vs D3 Group A | OR | 1.02 (0.73-1.42) | 0.926 | 1393 | 349 |
| MIMIC-IV | Post-day-3 hospital mortality | D3 Group B vs D3 Group A | OR | 1.25 (0.70-2.25) | 0.450 | 1393 | 349 |
| MIMIC-IV | Post-day-3 30-day mortality | D3 Group C vs D3 Group A | HR | 1.11 (0.85-1.44) | 0.445 | 1393 | 376 |
| MIMIC-IV | Post-day-3 30-day mortality | D3 Group B vs D3 Group A | HR | 1.16 (0.76-1.78) | 0.488 | 1393 | 376 |
| MIMIC-IV | Post-day-3 90-day mortality | D3 Group C vs D3 Group A | HR | 1.04 (0.82-1.33) | 0.737 | 1393 | 463 |
| MIMIC-IV | Post-day-3 90-day mortality | D3 Group B vs D3 Group A | HR | 1.19 (0.81-1.76) | 0.373 | 1393 | 463 |
| eICU | Post-day-3 hospital mortality | D3 Group C vs D3 Group A | OR | 1.15 (0.94-1.42) | 0.170 | 10184 | 1884 |
| eICU | Post-day-3 hospital mortality | D3 Group B vs D3 Group A | OR | 1.87 (1.48-2.37) | <0.001 | 10184 | 1884 |
| eICU | Post-day-3 30-day in-hospital mortality | D3 Group C vs D3 Group A | OR | 1.17 (0.95-1.44) | 0.132 | 10184 | 1809 |
| eICU | Post-day-3 30-day in-hospital mortality | D3 Group B vs D3 Group A | OR | 1.83 (1.45-2.33) | <0.001 | 10184 | 1809 |

Note. Trajectories were reconstructed using ICU days 1-3, and outcomes were analyzed among patients alive and still hospitalized at ICU day 3.

**Supplementary Table S9. Extended adjustment analyses.**

| **Database** | **Outcome** | **Model** | **Comparison** | **Measure** | **Estimate (95% CI)** | **P value** | **n** | **Events** |
| --- | --- | --- | --- | --- | --- | --- | --- | --- |
| MIMIC-IV | Hospital mortality | Model 4: original fully adjusted | Group C vs Group A | OR | 1.30 (0.95-1.78) | 0.097 | 1243 | 350 |
| MIMIC-IV | Hospital mortality | Model 4: original fully adjusted | Group B vs Group A | OR | 1.61 (0.98-2.64) | 0.058 | 1243 | 350 |
| MIMIC-IV | 30-day mortality | Model 4: original fully adjusted | Group C vs Group A | HR | 1.28 (1.00-1.65) | 0.052 | 1243 | 357 |
| MIMIC-IV | 30-day mortality | Model 4: original fully adjusted | Group B vs Group A | HR | 1.59 (1.11-2.27) | 0.011 | 1243 | 357 |
| MIMIC-IV | 90-day mortality | Model 4: original fully adjusted | Group C vs Group A | HR | 1.37 (1.10-1.71) | 0.006 | 1243 | 439 |
| MIMIC-IV | 90-day mortality | Model 4: original fully adjusted | Group B vs Group A | HR | 1.59 (1.14-2.21) | 0.006 | 1243 | 439 |
| eICU | Hospital mortality | Model 4: original fully adjusted | Group C vs Group A | OR | 1.29 (1.14-1.46) | <0.001 | 9090 | 1760 |
| eICU | Hospital mortality | Model 4: original fully adjusted | Group B vs Group A | OR | 1.95 (1.55-2.45) | <0.001 | 9090 | 1760 |
| eICU | 30-day in-hospital mortality | Model 4: original fully adjusted | Group C vs Group A | OR | 1.32 (1.16-1.50) | <0.001 | 9090 | 1674 |
| eICU | 30-day in-hospital mortality | Model 4: original fully adjusted | Group B vs Group A | OR | 1.93 (1.53-2.44) | <0.001 | 9090 | 1674 |
| MIMIC-IV | Hospital mortality | Model 4 + baseline GNRI | Group C vs Group A | OR | 1.69 (1.16-2.46) | 0.006 | 1202 | 337 |
| MIMIC-IV | Hospital mortality | Model 4 + baseline GNRI | Group B vs Group A | OR | 1.61 (0.98-2.66) | 0.061 | 1202 | 337 |
| MIMIC-IV | 30-day mortality | Model 4 + baseline GNRI | Group C vs Group A | HR | 1.43 (1.07-1.91) | 0.016 | 1202 | 345 |
| MIMIC-IV | 30-day mortality | Model 4 + baseline GNRI | Group B vs Group A | HR | 1.60 (1.12-2.29) | 0.010 | 1202 | 345 |
| MIMIC-IV | 90-day mortality | Model 4 + baseline GNRI | Group C vs Group A | HR | 1.62 (1.25-2.10) | <0.001 | 1202 | 425 |
| MIMIC-IV | 90-day mortality | Model 4 + baseline GNRI | Group B vs Group A | HR | 1.58 (1.13-2.20) | 0.007 | 1202 | 425 |
| eICU | Hospital mortality | Model 4 + baseline GNRI | Group C vs Group A | OR | 1.58 (1.37-1.81) | <0.001 | 8948 | 1733 |
| eICU | Hospital mortality | Model 4 + baseline GNRI | Group B vs Group A | OR | 1.77 (1.41-2.23) | <0.001 | 8948 | 1733 |
| eICU | 30-day in-hospital mortality | Model 4 + baseline GNRI | Group C vs Group A | OR | 1.58 (1.37-1.83) | <0.001 | 8948 | 1649 |
| eICU | 30-day in-hospital mortality | Model 4 + baseline GNRI | Group B vs Group A | OR | 1.77 (1.40-2.23) | <0.001 | 8948 | 1649 |
| MIMIC-IV | Hospital mortality | Model 4 + baseline albumin | Group C vs Group A | OR | 1.67 (1.15-2.44) | 0.007 | 1202 | 337 |
| MIMIC-IV | Hospital mortality | Model 4 + baseline albumin | Group B vs Group A | OR | 1.62 (0.98-2.67) | 0.060 | 1202 | 337 |
| MIMIC-IV | 30-day mortality | Model 4 + baseline albumin | Group C vs Group A | HR | 1.42 (1.06-1.90) | 0.018 | 1202 | 345 |
| MIMIC-IV | 30-day mortality | Model 4 + baseline albumin | Group B vs Group A | HR | 1.60 (1.12-2.29) | 0.010 | 1202 | 345 |
| MIMIC-IV | 90-day mortality | Model 4 + baseline albumin | Group C vs Group A | HR | 1.59 (1.22-2.06) | <0.001 | 1202 | 425 |
| MIMIC-IV | 90-day mortality | Model 4 + baseline albumin | Group B vs Group A | HR | 1.58 (1.14-2.21) | 0.007 | 1202 | 425 |
| eICU | Hospital mortality | Model 4 + baseline albumin | Group C vs Group A | OR | 1.54 (1.34-1.78) | <0.001 | 8948 | 1733 |
| eICU | Hospital mortality | Model 4 + baseline albumin | Group B vs Group A | OR | 1.78 (1.41-2.25) | <0.001 | 8948 | 1733 |
| eICU | 30-day in-hospital mortality | Model 4 + baseline albumin | Group C vs Group A | OR | 1.55 (1.34-1.79) | <0.001 | 8948 | 1649 |
| eICU | 30-day in-hospital mortality | Model 4 + baseline albumin | Group B vs Group A | OR | 1.78 (1.41-2.25) | <0.001 | 8948 | 1649 |
| MIMIC-IV | Hospital mortality | Model 4 + day-1 fluid balance | Group C vs Group A | OR | 1.28 (0.93-1.76) | 0.135 | 1224 | 340 |
| MIMIC-IV | Hospital mortality | Model 4 + day-1 fluid balance | Group B vs Group A | OR | 1.66 (1.01-2.72) | 0.045 | 1224 | 340 |
| MIMIC-IV | 30-day mortality | Model 4 + day-1 fluid balance | Group C vs Group A | HR | 1.25 (0.97-1.62) | 0.085 | 1224 | 346 |
| MIMIC-IV | 30-day mortality | Model 4 + day-1 fluid balance | Group B vs Group A | HR | 1.63 (1.14-2.34) | 0.007 | 1224 | 346 |
| MIMIC-IV | 90-day mortality | Model 4 + day-1 fluid balance | Group C vs Group A | HR | 1.35 (1.07-1.70) | 0.010 | 1224 | 427 |
| MIMIC-IV | 90-day mortality | Model 4 + day-1 fluid balance | Group B vs Group A | HR | 1.63 (1.17-2.26) | 0.004 | 1224 | 427 |
| eICU | Hospital mortality | Model 4 + day-1 fluid balance | Group C vs Group A | OR | 1.30 (1.14-1.47) | <0.001 | 8868 | 1730 |
| eICU | Hospital mortality | Model 4 + day-1 fluid balance | Group B vs Group A | OR | 1.96 (1.55-2.46) | <0.001 | 8868 | 1730 |
| eICU | 30-day in-hospital mortality | Model 4 + day-1 fluid balance | Group C vs Group A | OR | 1.32 (1.16-1.51) | <0.001 | 8868 | 1645 |
| eICU | 30-day in-hospital mortality | Model 4 + day-1 fluid balance | Group B vs Group A | OR | 1.94 (1.53-2.45) | <0.001 | 8868 | 1645 |
| MIMIC-IV | Hospital mortality | Model 4 + mean fluid balance days 1-3 | Group C vs Group A | OR | 1.32 (0.96-1.80) | 0.084 | 1243 | 350 |
| MIMIC-IV | Hospital mortality | Model 4 + mean fluid balance days 1-3 | Group B vs Group A | OR | 1.59 (0.97-2.61) | 0.065 | 1243 | 350 |
| MIMIC-IV | 30-day mortality | Model 4 + mean fluid balance days 1-3 | Group C vs Group A | HR | 1.29 (1.00-1.65) | 0.050 | 1243 | 357 |
| MIMIC-IV | 30-day mortality | Model 4 + mean fluid balance days 1-3 | Group B vs Group A | HR | 1.59 (1.11-2.27) | 0.011 | 1243 | 357 |
| MIMIC-IV | 90-day mortality | Model 4 + mean fluid balance days 1-3 | Group C vs Group A | HR | 1.37 (1.10-1.72) | 0.005 | 1243 | 439 |
| MIMIC-IV | 90-day mortality | Model 4 + mean fluid balance days 1-3 | Group B vs Group A | HR | 1.58 (1.14-2.20) | 0.006 | 1243 | 439 |
| eICU | Hospital mortality | Model 4 + mean fluid balance days 1-3 | Group C vs Group A | OR | 1.28 (1.13-1.45) | <0.001 | 9066 | 1760 |
| eICU | Hospital mortality | Model 4 + mean fluid balance days 1-3 | Group B vs Group A | OR | 1.95 (1.55-2.46) | <0.001 | 9066 | 1760 |
| eICU | 30-day in-hospital mortality | Model 4 + mean fluid balance days 1-3 | Group C vs Group A | OR | 1.30 (1.15-1.48) | <0.001 | 9066 | 1674 |
| eICU | 30-day in-hospital mortality | Model 4 + mean fluid balance days 1-3 | Group B vs Group A | OR | 1.94 (1.54-2.44) | <0.001 | 9066 | 1674 |
| MIMIC-IV | Hospital mortality | Model 4 + cumulative fluid balance days 1-3 | Group C vs Group A | OR | 1.31 (0.96-1.79) | 0.092 | 1243 | 350 |
| MIMIC-IV | Hospital mortality | Model 4 + cumulative fluid balance days 1-3 | Group B vs Group A | OR | 1.60 (0.98-2.63) | 0.061 | 1243 | 350 |
| MIMIC-IV | 30-day mortality | Model 4 + cumulative fluid balance days 1-3 | Group C vs Group A | HR | 1.28 (1.00-1.65) | 0.052 | 1243 | 357 |
| MIMIC-IV | 30-day mortality | Model 4 + cumulative fluid balance days 1-3 | Group B vs Group A | HR | 1.59 (1.11-2.27) | 0.011 | 1243 | 357 |
| MIMIC-IV | 90-day mortality | Model 4 + cumulative fluid balance days 1-3 | Group C vs Group A | HR | 1.37 (1.09-1.71) | 0.006 | 1243 | 439 |
| MIMIC-IV | 90-day mortality | Model 4 + cumulative fluid balance days 1-3 | Group B vs Group A | HR | 1.59 (1.14-2.21) | 0.006 | 1243 | 439 |
| eICU | Hospital mortality | Model 4 + cumulative fluid balance days 1-3 | Group C vs Group A | OR | 1.28 (1.13-1.45) | <0.001 | 9090 | 1760 |
| eICU | Hospital mortality | Model 4 + cumulative fluid balance days 1-3 | Group B vs Group A | OR | 1.95 (1.55-2.45) | <0.001 | 9090 | 1760 |
| eICU | 30-day in-hospital mortality | Model 4 + cumulative fluid balance days 1-3 | Group C vs Group A | OR | 1.31 (1.15-1.49) | <0.001 | 9090 | 1674 |
| eICU | 30-day in-hospital mortality | Model 4 + cumulative fluid balance days 1-3 | Group B vs Group A | OR | 1.93 (1.53-2.43) | <0.001 | 9090 | 1674 |
| MIMIC-IV | Hospital mortality | Model 4 + GNRI + albumin + day-1 fluid + cumulative fluid days 1-3 | Group C vs Group A | OR | 1.59 (1.07-2.34) | 0.020 | 1185 | 328 |
| MIMIC-IV | Hospital mortality | Model 4 + GNRI + albumin + day-1 fluid + cumulative fluid days 1-3 | Group B vs Group A | OR | 1.64 (0.99-2.72) | 0.055 | 1185 | 328 |
| MIMIC-IV | 30-day mortality | Model 4 + GNRI + albumin + day-1 fluid + cumulative fluid days 1-3 | Group C vs Group A | HR | 1.37 (1.01-1.86) | 0.044 | 1185 | 335 |
| MIMIC-IV | 30-day mortality | Model 4 + GNRI + albumin + day-1 fluid + cumulative fluid days 1-3 | Group B vs Group A | HR | 1.64 (1.14-2.35) | 0.008 | 1185 | 335 |
| MIMIC-IV | 90-day mortality | Model 4 + GNRI + albumin + day-1 fluid + cumulative fluid days 1-3 | Group C vs Group A | HR | 1.55 (1.18-2.04) | 0.002 | 1185 | 414 |
| MIMIC-IV | 90-day mortality | Model 4 + GNRI + albumin + day-1 fluid + cumulative fluid days 1-3 | Group B vs Group A | HR | 1.61 (1.15-2.24) | 0.005 | 1185 | 414 |
| eICU | Hospital mortality | Model 4 + GNRI + albumin + day-1 fluid + cumulative fluid days 1-3 | Group C vs Group A | OR | 1.51 (1.31-1.74) | <0.001 | 8730 | 1704 |
| eICU | Hospital mortality | Model 4 + GNRI + albumin + day-1 fluid + cumulative fluid days 1-3 | Group B vs Group A | OR | 1.83 (1.44-2.31) | <0.001 | 8730 | 1704 |
| eICU | 30-day in-hospital mortality | Model 4 + GNRI + albumin + day-1 fluid + cumulative fluid days 1-3 | Group C vs Group A | OR | 1.52 (1.31-1.76) | <0.001 | 8730 | 1620 |
| eICU | 30-day in-hospital mortality | Model 4 + GNRI + albumin + day-1 fluid + cumulative fluid days 1-3 | Group B vs Group A | OR | 1.82 (1.44-2.31) | <0.001 | 8730 | 1620 |

Note. Model 4 was additionally adjusted for baseline GNRI, baseline albumin, day-1 fluid balance, mean fluid balance during ICU days 1-3, cumulative fluid balance during ICU days 1-3, or the combined extended adjustment set. Continuous added variables were standardized before modeling.

**Supplementary Table S10. Incremental prediction performance.**

| **Database** | **Outcome** | **Model** | **n** | **Events** | **AUC (95% CI)** | **Brier score** | **Calibration intercept** | **Calibration slope** | **Calibration intercept (slope=1)** |
| --- | --- | --- | --- | --- | --- | --- | --- | --- | --- |
| MIMIC-IV | Hospital mortality | Base | 1185 | 328 | 0.674 (0.639-0.710) | 0.186 | -0.142 | 0.836 | -0.001 |
| MIMIC-IV | Hospital mortality | Base + GNRI | 1185 | 328 | 0.678 (0.642-0.714) | 0.185 | -0.126 | 0.849 | 0.005 |
| MIMIC-IV | Hospital mortality | Base + fluid | 1185 | 328 | 0.693 (0.658-0.728) | 0.183 | -0.127 | 0.849 | 0.001 |
| MIMIC-IV | Hospital mortality | Base + GNRI + fluid | 1185 | 328 | 0.697 (0.662-0.732) | 0.182 | -0.118 | 0.86 | 0.0 |
| MIMIC-IV | Hospital mortality | Base + trajectory | 1185 | 328 | 0.677 (0.642-0.713) | 0.185 | -0.139 | 0.836 | 0.003 |
| MIMIC-IV | Hospital mortality | Base + GNRI + fluid + trajectory | 1185 | 328 | 0.695 (0.660-0.731) | 0.183 | -0.147 | 0.817 | 0.01 |
| MIMIC-IV | 30-day mortality | Base | 1185 | 335 | 0.671 (0.635-0.706) | 0.189 | -0.144 | 0.82 | 0.008 |
| MIMIC-IV | 30-day mortality | Base + GNRI | 1185 | 335 | 0.675 (0.640-0.711) | 0.188 | -0.135 | 0.843 | -0.004 |
| MIMIC-IV | 30-day mortality | Base + fluid | 1185 | 335 | 0.686 (0.651-0.722) | 0.187 | -0.167 | 0.801 | -0.003 |
| MIMIC-IV | 30-day mortality | Base + GNRI + fluid | 1185 | 335 | 0.685 (0.650-0.720) | 0.187 | -0.17 | 0.796 | -0.002 |
| MIMIC-IV | 30-day mortality | Base + trajectory | 1185 | 335 | 0.686 (0.651-0.721) | 0.185 | -0.085 | 0.896 | 0.002 |
| MIMIC-IV | 30-day mortality | Base + GNRI + fluid + trajectory | 1185 | 335 | 0.701 (0.666-0.736) | 0.183 | -0.122 | 0.853 | -0.001 |
| MIMIC-IV | 90-day mortality | Base | 1185 | 414 | 0.702 (0.670-0.734) | 0.202 | -0.069 | 0.889 | -0.011 |
| MIMIC-IV | 90-day mortality | Base + GNRI | 1185 | 414 | 0.703 (0.670-0.735) | 0.202 | -0.066 | 0.882 | -0.002 |
| MIMIC-IV | 90-day mortality | Base + fluid | 1185 | 414 | 0.720 (0.689-0.752) | 0.197 | -0.049 | 0.903 | 0.004 |
| MIMIC-IV | 90-day mortality | Base + GNRI + fluid | 1185 | 414 | 0.725 (0.693-0.756) | 0.196 | -0.041 | 0.918 | 0.003 |
| MIMIC-IV | 90-day mortality | Base + trajectory | 1185 | 414 | 0.704 (0.672-0.737) | 0.201 | -0.063 | 0.884 | -0.0 |
| MIMIC-IV | 90-day mortality | Base + GNRI + fluid + trajectory | 1185 | 414 | 0.725 (0.693-0.756) | 0.196 | -0.071 | 0.871 | -0.003 |
| eICU | Hospital mortality | Base | 8730 | 1704 | 0.692 (0.677-0.707) | 0.146 | -0.032 | 0.974 | 0.001 |
| eICU | Hospital mortality | Base + GNRI | 8730 | 1704 | 0.695 (0.680-0.710) | 0.145 | -0.026 | 0.98 | -0.0 |
| eICU | Hospital mortality | Base + fluid | 8730 | 1704 | 0.699 (0.684-0.714) | 0.145 | -0.033 | 0.975 | -0.001 |
| eICU | Hospital mortality | Base + GNRI + fluid | 8730 | 1704 | 0.702 (0.688-0.717) | 0.144 | -0.03 | 0.977 | -0.001 |
| eICU | Hospital mortality | Base + trajectory | 8730 | 1704 | 0.698 (0.683-0.713) | 0.145 | -0.027 | 0.979 | 0.0 |
| eICU | Hospital mortality | Base + GNRI + fluid + trajectory | 8730 | 1704 | 0.707 (0.693-0.722) | 0.144 | -0.046 | 0.965 | -0.001 |
| eICU | 30-day in-hospital mortality | Base | 8730 | 1620 | 0.694 (0.679-0.710) | 0.141 | -0.029 | 0.978 | -0.001 |
| eICU | 30-day in-hospital mortality | Base + GNRI | 8730 | 1620 | 0.695 (0.680-0.711) | 0.14 | -0.033 | 0.974 | 0.001 |
| eICU | 30-day in-hospital mortality | Base + fluid | 8730 | 1620 | 0.700 (0.685-0.715) | 0.14 | -0.036 | 0.972 | 0.002 |
| eICU | 30-day in-hospital mortality | Base + GNRI + fluid | 8730 | 1620 | 0.700 (0.685-0.715) | 0.14 | -0.052 | 0.96 | 0.002 |
| eICU | 30-day in-hospital mortality | Base + trajectory | 8730 | 1620 | 0.699 (0.683-0.714) | 0.14 | -0.041 | 0.969 | -0.0 |
| eICU | 30-day in-hospital mortality | Base + GNRI + fluid + trajectory | 8730 | 1620 | 0.710 (0.694-0.725) | 0.138 | -0.037 | 0.973 | -0.001 |

Note. Predictive performance was evaluated using 5-fold cross-validated predicted probabilities. AUC, Brier score, and calibration metrics are reported for complete-case prediction datasets.

**Supplementary Table S11. Incremental model comparison statistics.**

**Panel A. Delta AUC, delta Brier score, and likelihood ratio test.**

| **Database** | **Outcome** | **Comparison** | **n** | **Events** | **AUC old** | **AUC new** | **Delta AUC** | **Brier old** | **Brier new** | **Delta Brier** | **LR P value** |
| --- | --- | --- | --- | --- | --- | --- | --- | --- | --- | --- | --- |
| MIMIC-IV | Hospital mortality | Base + baseline GNRI vs Base covariates | 1185 | 328 | 0.674 | 0.678 | 0.004 | 0.186 | 0.185 | -0.001 | 0.208 |
| MIMIC-IV | Hospital mortality | Base + early fluid balance vs Base covariates | 1185 | 328 | 0.674 | 0.693 | 0.018 | 0.186 | 0.183 | -0.004 | <0.001 |
| MIMIC-IV | Hospital mortality | Base + GNRI + early fluid vs Base covariates | 1185 | 328 | 0.674 | 0.697 | 0.023 | 0.186 | 0.182 | -0.004 | <0.001 |
| MIMIC-IV | Hospital mortality | Base + joint trajectory vs Base covariates | 1185 | 328 | 0.674 | 0.677 | 0.003 | 0.186 | 0.185 | -0.001 | 0.035 |
| MIMIC-IV | Hospital mortality | Base + GNRI + early fluid + joint trajectory vs Base covariates | 1185 | 328 | 0.674 | 0.695 | 0.021 | 0.186 | 0.183 | -0.003 | <0.001 |
| MIMIC-IV | Hospital mortality | Base + GNRI + early fluid + joint trajectory vs Base + GNRI + early fluid | 1185 | 328 | 0.697 | 0.695 | -0.002 | 0.182 | 0.183 | 0.001 | 0.016 |
| MIMIC-IV | 30-day mortality | Base + baseline GNRI vs Base covariates | 1185 | 335 | 0.671 | 0.675 | 0.005 | 0.189 | 0.188 | -0.001 | 0.436 |
| MIMIC-IV | 30-day mortality | Base + early fluid balance vs Base covariates | 1185 | 335 | 0.671 | 0.686 | 0.016 | 0.189 | 0.187 | -0.002 | <0.001 |
| MIMIC-IV | 30-day mortality | Base + GNRI + early fluid vs Base covariates | 1185 | 335 | 0.671 | 0.685 | 0.015 | 0.189 | 0.187 | -0.002 | <0.001 |
| MIMIC-IV | 30-day mortality | Base + joint trajectory vs Base covariates | 1185 | 335 | 0.671 | 0.686 | 0.016 | 0.189 | 0.185 | -0.004 | 0.025 |
| MIMIC-IV | 30-day mortality | Base + GNRI + early fluid + joint trajectory vs Base covariates | 1185 | 335 | 0.671 | 0.701 | 0.03 | 0.189 | 0.183 | -0.006 | <0.001 |
| MIMIC-IV | 30-day mortality | Base + GNRI + early fluid + joint trajectory vs Base + GNRI + early fluid | 1185 | 335 | 0.685 | 0.701 | 0.016 | 0.187 | 0.183 | -0.004 | 0.019 |
| MIMIC-IV | 90-day mortality | Base + baseline GNRI vs Base covariates | 1185 | 414 | 0.702 | 0.703 | 0.001 | 0.202 | 0.202 | -0.0 | 0.192 |
| MIMIC-IV | 90-day mortality | Base + early fluid balance vs Base covariates | 1185 | 414 | 0.702 | 0.72 | 0.019 | 0.202 | 0.197 | -0.005 | <0.001 |
| MIMIC-IV | 90-day mortality | Base + GNRI + early fluid vs Base covariates | 1185 | 414 | 0.702 | 0.725 | 0.023 | 0.202 | 0.196 | -0.006 | <0.001 |
| MIMIC-IV | 90-day mortality | Base + joint trajectory vs Base covariates | 1185 | 414 | 0.702 | 0.704 | 0.003 | 0.202 | 0.201 | -0.001 | 0.004 |
| MIMIC-IV | 90-day mortality | Base + GNRI + early fluid + joint trajectory vs Base covariates | 1185 | 414 | 0.702 | 0.725 | 0.023 | 0.202 | 0.196 | -0.006 | <0.001 |
| MIMIC-IV | 90-day mortality | Base + GNRI + early fluid + joint trajectory vs Base + GNRI + early fluid | 1185 | 414 | 0.725 | 0.725 | 0.0 | 0.196 | 0.196 | -0.0 | <0.001 |
| eICU | Hospital mortality | Base + baseline GNRI vs Base covariates | 8730 | 1704 | 0.692 | 0.695 | 0.003 | 0.146 | 0.145 | -0.0 | <0.001 |
| eICU | Hospital mortality | Base + early fluid balance vs Base covariates | 8730 | 1704 | 0.692 | 0.699 | 0.007 | 0.146 | 0.145 | -0.001 | <0.001 |
| eICU | Hospital mortality | Base + GNRI + early fluid vs Base covariates | 8730 | 1704 | 0.692 | 0.702 | 0.011 | 0.146 | 0.144 | -0.002 | <0.001 |
| eICU | Hospital mortality | Base + joint trajectory vs Base covariates | 8730 | 1704 | 0.692 | 0.698 | 0.006 | 0.146 | 0.145 | -0.001 | <0.001 |
| eICU | Hospital mortality | Base + GNRI + early fluid + joint trajectory vs Base covariates | 8730 | 1704 | 0.692 | 0.707 | 0.016 | 0.146 | 0.144 | -0.002 | <0.001 |
| eICU | Hospital mortality | Base + GNRI + early fluid + joint trajectory vs Base + GNRI + early fluid | 8730 | 1704 | 0.702 | 0.707 | 0.005 | 0.144 | 0.144 | -0.001 | <0.001 |
| eICU | 30-day in-hospital mortality | Base + baseline GNRI vs Base covariates | 8730 | 1620 | 0.694 | 0.695 | 0.001 | 0.141 | 0.14 | -0.0 | <0.001 |
| eICU | 30-day in-hospital mortality | Base + early fluid balance vs Base covariates | 8730 | 1620 | 0.694 | 0.7 | 0.005 | 0.141 | 0.14 | -0.001 | <0.001 |
| eICU | 30-day in-hospital mortality | Base + GNRI + early fluid vs Base covariates | 8730 | 1620 | 0.694 | 0.7 | 0.005 | 0.141 | 0.14 | -0.001 | <0.001 |
| eICU | 30-day in-hospital mortality | Base + joint trajectory vs Base covariates | 8730 | 1620 | 0.694 | 0.699 | 0.004 | 0.141 | 0.14 | -0.001 | <0.001 |
| eICU | 30-day in-hospital mortality | Base + GNRI + early fluid + joint trajectory vs Base covariates | 8730 | 1620 | 0.694 | 0.71 | 0.015 | 0.141 | 0.138 | -0.002 | <0.001 |
| eICU | 30-day in-hospital mortality | Base + GNRI + early fluid + joint trajectory vs Base + GNRI + early fluid | 8730 | 1620 | 0.7 | 0.71 | 0.01 | 0.14 | 0.138 | -0.001 | <0.001 |

**Panel B. IDI and continuous NRI.**

| **Database** | **Outcome** | **Comparison** | **IDI** | **Continuous NRI** | **NRI event component** | **NRI nonevent component** |
| --- | --- | --- | --- | --- | --- | --- |
| MIMIC-IV | Hospital mortality | Base + baseline GNRI vs Base covariates | 0.003 | 0.072 | 0.055 | 0.018 |
| MIMIC-IV | Hospital mortality | Base + early fluid balance vs Base covariates | 0.019 | 0.256 | 0.122 | 0.134 |
| MIMIC-IV | Hospital mortality | Base + GNRI + early fluid vs Base covariates | 0.021 | 0.3 | 0.14 | 0.16 |
| MIMIC-IV | Hospital mortality | Base + joint trajectory vs Base covariates | 0.006 | 0.117 | -0.043 | 0.16 |
| MIMIC-IV | Hospital mortality | Base + GNRI + early fluid + joint trajectory vs Base covariates | 0.023 | 0.262 | 0.085 | 0.176 |
| MIMIC-IV | Hospital mortality | Base + GNRI + early fluid + joint trajectory vs Base + GNRI + early fluid | 0.002 | 0.05 | -0.079 | 0.13 |
| MIMIC-IV | 30-day mortality | Base + baseline GNRI vs Base covariates | 0.002 | 0.012 | 0.057 | -0.045 |
| MIMIC-IV | 30-day mortality | Base + early fluid balance vs Base covariates | 0.015 | 0.233 | 0.176 | 0.056 |
| MIMIC-IV | 30-day mortality | Base + GNRI + early fluid vs Base covariates | 0.016 | 0.156 | 0.104 | 0.052 |
| MIMIC-IV | 30-day mortality | Base + joint trajectory vs Base covariates | 0.013 | 0.207 | 0.045 | 0.162 |
| MIMIC-IV | 30-day mortality | Base + GNRI + early fluid + joint trajectory vs Base covariates | 0.028 | 0.285 | 0.158 | 0.127 |
| MIMIC-IV | 30-day mortality | Base + GNRI + early fluid + joint trajectory vs Base + GNRI + early fluid | 0.012 | 0.122 | 0.009 | 0.113 |
| MIMIC-IV | 90-day mortality | Base + baseline GNRI vs Base covariates | -0.001 | -0.03 | -0.034 | 0.004 |
| MIMIC-IV | 90-day mortality | Base + early fluid balance vs Base covariates | 0.018 | 0.203 | 0.072 | 0.131 |
| MIMIC-IV | 90-day mortality | Base + GNRI + early fluid vs Base covariates | 0.022 | 0.28 | 0.155 | 0.126 |
| MIMIC-IV | 90-day mortality | Base + joint trajectory vs Base covariates | 0.006 | 0.12 | -0.126 | 0.245 |
| MIMIC-IV | 90-day mortality | Base + GNRI + early fluid + joint trajectory vs Base covariates | 0.029 | 0.289 | 0.106 | 0.183 |
| MIMIC-IV | 90-day mortality | Base + GNRI + early fluid + joint trajectory vs Base + GNRI + early fluid | 0.007 | 0.179 | -0.014 | 0.193 |
| eICU | Hospital mortality | Base + baseline GNRI vs Base covariates | 0.002 | 0.134 | 0.076 | 0.058 |
| eICU | Hospital mortality | Base + early fluid balance vs Base covariates | 0.007 | 0.199 | 0.14 | 0.06 |
| eICU | Hospital mortality | Base + GNRI + early fluid vs Base covariates | 0.01 | 0.217 | 0.108 | 0.109 |
| eICU | Hospital mortality | Base + joint trajectory vs Base covariates | 0.006 | 0.136 | -0.204 | 0.34 |
| eICU | Hospital mortality | Base + GNRI + early fluid + joint trajectory vs Base covariates | 0.015 | 0.279 | 0.11 | 0.169 |
| eICU | Hospital mortality | Base + GNRI + early fluid + joint trajectory vs Base + GNRI + early fluid | 0.005 | 0.14 | -0.18 | 0.32 |
| eICU | 30-day in-hospital mortality | Base + baseline GNRI vs Base covariates | 0.001 | 0.1 | 0.027 | 0.073 |
| eICU | 30-day in-hospital mortality | Base + early fluid balance vs Base covariates | 0.005 | 0.168 | 0.091 | 0.077 |
| eICU | 30-day in-hospital mortality | Base + GNRI + early fluid vs Base covariates | 0.006 | 0.186 | 0.068 | 0.118 |
| eICU | 30-day in-hospital mortality | Base + joint trajectory vs Base covariates | 0.005 | 0.126 | -0.201 | 0.328 |
| eICU | 30-day in-hospital mortality | Base + GNRI + early fluid + joint trajectory vs Base covariates | 0.014 | 0.258 | 0.101 | 0.157 |
| eICU | 30-day in-hospital mortality | Base + GNRI + early fluid + joint trajectory vs Base + GNRI + early fluid | 0.008 | 0.156 | -0.13 | 0.285 |

**Supplementary Table S12. Proportional hazards assumption tests for MIMIC-IV Cox models.**

| **Database** | **Outcome** | **n** | **Events** | **Term** | **Test** | **Chi-square** | **df** | **P value** | **Interpretation** |
| --- | --- | --- | --- | --- | --- | --- | --- | --- | --- |
| MIMIC-IV | 30-day mortality | 1243 | 357 | joint_traj_group | Schoenfeld residuals | 9.662 | 2 | 0.008 | Potential violation |
| MIMIC-IV | 30-day mortality | 1243 | 357 | age | Schoenfeld residuals | 5.108 | 1 | 0.024 | Potential violation |
| MIMIC-IV | 30-day mortality | 1243 | 357 | sex | Schoenfeld residuals | 2.909 | 1 | 0.088 | No evidence of violation |
| MIMIC-IV | 30-day mortality | 1243 | 357 | bmi | Schoenfeld residuals | 0.042 | 1 | 0.837 | No evidence of violation |
| MIMIC-IV | 30-day mortality | 1243 | 357 | sofa | Schoenfeld residuals | 0.534 | 1 | 0.465 | No evidence of violation |
| MIMIC-IV | 30-day mortality | 1243 | 357 | charlson | Schoenfeld residuals | 8.637 | 1 | 0.003 | Potential violation |
| MIMIC-IV | 30-day mortality | 1243 | 357 | ckd | Schoenfeld residuals | 3.006 | 1 | 0.083 | No evidence of violation |
| MIMIC-IV | 30-day mortality | 1243 | 357 | hf | Schoenfeld residuals | 0.24 | 1 | 0.624 | No evidence of violation |
| MIMIC-IV | 30-day mortality | 1243 | 357 | sepsis | Schoenfeld residuals | 1.571 | 1 | 0.210 | No evidence of violation |
| MIMIC-IV | 30-day mortality | 1243 | 357 | ventilation_24h | Schoenfeld residuals | 0.006 | 1 | 0.940 | No evidence of violation |
| MIMIC-IV | 30-day mortality | 1243 | 357 | rrt_24h | Schoenfeld residuals | 0.572 | 1 | 0.449 | No evidence of violation |
| MIMIC-IV | 30-day mortality | 1243 | 357 | furosemide_24h | Schoenfeld residuals | 0.019 | 1 | 0.891 | No evidence of violation |
| MIMIC-IV | 30-day mortality | 1243 | 357 | spironolactone_24h | Schoenfeld residuals | 0.271 | 1 | 0.603 | No evidence of violation |
| MIMIC-IV | 30-day mortality | 1243 | 357 | norepinephrine_24h | Schoenfeld residuals | 0.094 | 1 | 0.760 | No evidence of violation |
| MIMIC-IV | 30-day mortality | 1243 | 357 | GLOBAL | Schoenfeld residuals | 29.147 | 15 | 0.015 | Potential violation |
| MIMIC-IV | 90-day mortality | 1243 | 439 | joint_traj_group | Schoenfeld residuals | 3.248 | 2 | 0.197 | No evidence of violation |
| MIMIC-IV | 90-day mortality | 1243 | 439 | age | Schoenfeld residuals | 3.379 | 1 | 0.066 | No evidence of violation |
| MIMIC-IV | 90-day mortality | 1243 | 439 | sex | Schoenfeld residuals | 1.661 | 1 | 0.197 | No evidence of violation |
| MIMIC-IV | 90-day mortality | 1243 | 439 | bmi | Schoenfeld residuals | 0.159 | 1 | 0.690 | No evidence of violation |
| MIMIC-IV | 90-day mortality | 1243 | 439 | sofa | Schoenfeld residuals | 1.594 | 1 | 0.207 | No evidence of violation |
| MIMIC-IV | 90-day mortality | 1243 | 439 | charlson | Schoenfeld residuals | 19.441 | 1 | <0.001 | Potential violation |
| MIMIC-IV | 90-day mortality | 1243 | 439 | ckd | Schoenfeld residuals | 5.792 | 1 | 0.016 | Potential violation |
| MIMIC-IV | 90-day mortality | 1243 | 439 | hf | Schoenfeld residuals | 0.344 | 1 | 0.557 | No evidence of violation |
| MIMIC-IV | 90-day mortality | 1243 | 439 | sepsis | Schoenfeld residuals | 1.288 | 1 | 0.256 | No evidence of violation |
| MIMIC-IV | 90-day mortality | 1243 | 439 | ventilation_24h | Schoenfeld residuals | 1.189 | 1 | 0.275 | No evidence of violation |
| MIMIC-IV | 90-day mortality | 1243 | 439 | rrt_24h | Schoenfeld residuals | 0.06 | 1 | 0.806 | No evidence of violation |
| MIMIC-IV | 90-day mortality | 1243 | 439 | furosemide_24h | Schoenfeld residuals | 0.2 | 1 | 0.655 | No evidence of violation |
| MIMIC-IV | 90-day mortality | 1243 | 439 | spironolactone_24h | Schoenfeld residuals | 1.812 | 1 | 0.178 | No evidence of violation |
| MIMIC-IV | 90-day mortality | 1243 | 439 | norepinephrine_24h | Schoenfeld residuals | 1.751 | 1 | 0.186 | No evidence of violation |
| MIMIC-IV | 90-day mortality | 1243 | 439 | GLOBAL | Schoenfeld residuals | 34.081 | 15 | 0.003 | Potential violation |

Note. The proportional hazards assumption was evaluated using Schoenfeld residuals. Potential non-proportionality was observed for the trajectory-group term in the 30-day Cox model and for the global tests; therefore, Cox-based estimates should be interpreted cautiously alongside fixed-time and landmark sensitivity analyses.

**Supplementary Table S13. High-confidence posterior classification sensitivity analysis.**

| **Database** | **Outcome** | **Model type** | **n** | **Events** | **Comparison** | **Measure** | **Estimate (95% CI)** | **P value** |
| --- | --- | --- | --- | --- | --- | --- | --- | --- |
| MIMIC-IV | Hospital mortality | Logistic | 962 | 270 | Group C vs Group A | OR | 1.40 (0.96-2.05) | 0.077 |
| MIMIC-IV | Hospital mortality | Logistic | 962 | 270 | Group B vs Group A | OR | 2.12 (1.24-3.61) | 0.006 |
| MIMIC-IV | 30-day mortality | Cox | 962 | 277 | Group C vs Group A | HR | 1.23 (0.91-1.67) | 0.175 |
| MIMIC-IV | 30-day mortality | Cox | 962 | 277 | Group B vs Group A | HR | 1.93 (1.33-2.80) | <0.001 |
| MIMIC-IV | 90-day mortality | Cox | 962 | 347 | Group C vs Group A | HR | 1.37 (1.05-1.79) | 0.019 |
| MIMIC-IV | 90-day mortality | Cox | 962 | 347 | Group B vs Group A | HR | 1.87 (1.33-2.64) | <0.001 |
| eICU | Hospital mortality | Logistic | 7256 | 1408 | Group C vs Group A | OR | 1.31 (1.13-1.51) | <0.001 |
| eICU | Hospital mortality | Logistic | 7256 | 1408 | Group B vs Group A | OR | 1.93 (1.51-2.47) | <0.001 |
| eICU | 30-day in-hospital mortality | Logistic | 7256 | 1333 | Group C vs Group A | OR | 1.35 (1.16-1.56) | <0.001 |
| eICU | 30-day in-hospital mortality | Logistic | 7256 | 1333 | Group B vs Group A | OR | 1.93 (1.50-2.48) | <0.001 |

Note. This sensitivity analysis was restricted to patients with posterior probability >=0.70 for their assigned trajectory class. It evaluates the robustness of the modal class assignment approach, but it does not replace a formal three-step or probability-weighted latent-class outcome model.
